# The long non-coding RNA keratin-7 antisense acts as a new tumor suppressor to inhibit tumorigenesis and enhance apoptosis in lung and breast cancers

**DOI:** 10.1038/s41419-023-05802-3

**Published:** 2023-04-25

**Authors:** Zhe Zhao, Mei Meng, Jun Yao, Hao Zhou, Yu Chen, Juntao Liu, Jie Wang, Yuxi Liu, Yingnan Qiao, Mengli Zhang, Jindan Qi, Tong Zhang, Zhou Zhou, Tao Jiang, Bingxue Shang, Quansheng Zhou

**Affiliations:** 1 Cyrus Tang Hematology Center, Jiangsu Institute of Hematology, Soochow University, Suzhou, Jiangsu 215123 PR China; 2 CAS Key Laboratory of Nano-Bio Interface, Suzhou Institute of Nano-Tech and Nano-Bionics, Chinese Academy of Sciences, Suzhou, Jiangsu 215123 PR China; 3 Department of General Surgery, Dushu Lake Hospital Affiliated to Soochow University, Suzhou, Jiangsu 215123 PR China; 4 Department of General Surgery, The First Affiliated Hospital of Soochow University, Suzhou, Jiangsu 215006 PR China; 5 School of Nursing, Soochow University, Suzhou, Jiangsu 215006 PR China; 6 Department of Pathology, Dushu Lake Hospital Affiliated to Soochow University, Suzhou, Jiangsu 215123 PR China; 7 Institutes for Translational Medicine, State Key Laboratory of Radiation Medicine and Protection, Soochow University, 215123 Suzhou, PR China; 8 State Key Laboratory of Radiation Medicine and Protection, School of Radiation Medicine and Protection, Soochow University, Suzhou, Jiangsu 215123 PR China; 9 The First Affiliated Hospital of Soochow University, Suzhou, PR China; 10 National Clinical Research Center for Hematologic Diseases, The Affiliated Hospital of Soochow University, Suzhou, PR China; 11 Key Laboratory of Thrombosis and Hemostasis, Ministry of Health; Soochow University, Suzhou, Jiangsu 215123 PR China; 12 2011 Collaborative Innovation Center of Hematology, Soochow University, Suzhou, Jiangsu 215123 PR China; 13 The Ninth Affiliated Hospital, Soochow University, Suzhou, Jiangsu 215123 PR China

**Keywords:** Cell biology, Molecular biology, Lung cancer

## Abstract

Expression of the long non-coding RNA (lncRNA) keratin-7 antisense (KRT7-AS) is downregulated in various types of cancer; however, the impact of KRT7-AS deficiency on tumorigenesis and apoptosis is enigmatic. We aim to explore the influence of KRT7-AS in carcinogenesis and apoptosis. We found that KRT7-AS was deficient in breast and lung cancers, and low levels of KRT7-AS were a poor prognostic factor in breast cancer. Cellular studies showed that silencing of KRT7-AS in lung cancer cells increased oncogenic Keratin-7 levels and enhanced tumorigenesis, but diminished cancer apoptosis of the cancer cells; by contrast, overexpression of KRT7-AS inhibited lung cancer cell tumorigenesis. Additionally, KRT7-AS sensitized cancer cells to the anti-cancer drug cisplatin, consequently enhancing cancer cell apoptosis. In vivo, KRT7-AS overexpression significantly suppressed tumor growth in xenograft mice, while silencing of KRT7-AS promoted tumor growth. Mechanistically, KRT7-AS reduced the levels of oncogenic Keratin-7 and significantly elevated amounts of the key tumor suppressor PTEN in cancer cells through directly binding to PTEN protein via its core nucleic acid motif GGCAAUGGCGG. This inhibited the ubiquitination-proteasomal degradation of PTEN protein, therefore elevating PTEN levels in cancer cells. We also found that *KRT7-AS* gene transcription was driven by the transcription factor RXRα; intriguingly, the small molecule berberine enhanced KRT7-AS expression, reduced tumorigenesis, and promoted apoptosis of cancer cells. Collectively, KRT7-AS functions as a new tumor suppressor and an apoptosis enhancer in lung and breast cancers, and we unraveled that the RXRα-KRT7-AS-PTEN signaling axis controls carcinogenesis and apoptosis. Our findings highlight a tumor suppressive role of endogenous KRT7-AS in cancers and an important effect the RXRα-KRT7-AS-PTEN axis on control of cancer cell tumorigenesis and apoptosis, and offer a new platform for developing novel therapeutics against cancers.

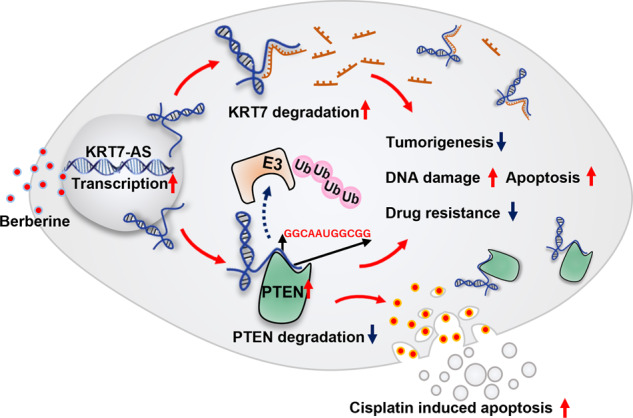

## Introduction

Numerous long non-coding RNAs (lncRNAs) have been shown to be involved in tumorigenesis [1–3] and apoptosis in cancer cells [4–10]. Several important tumor-suppressive and pro-apoptotic genes, such as p53 and PTEN, are mutated and/or downregulated in various types of cancer [11, 12]; on the other hand, numerous pro-apoptotic genes are deficient in cancers [8, 9, 13–16], resulting apoptosis resistance and uncontrolled tumor growth. It is of great interest to identify new endogenous tumor-suppressive and pro-apoptotic lncRNAs that control tumorigenesis and apoptosis.

Keratin-7 antisense (KRT7-AS) is a lncRNA that consists of 1698 nucleic acids and appears to have divergent roles in various types of cancer. KRT7-AS was reported to enhance tumorigenesis in gastric [17] and colorectal [18] cancers by stabilizing Keratin-7 (KRT7) mRNA; however, stabilization of KRT7 and KRT7-AS duplex was reported to promote lung metastasis in breast cancer [19]. Accumulated evidence shows that KRT7 is aberrantly overexpressed in various malignancies, including pancreatic cancer [20–22], bladder cancer [23], ovarian cancer [24–26], and various other malignant tumors [27–37]. Overexpression of KRT7 promotes tumorigenesis [28] and cancer metastasis [19], enhances drug resistance, and induces apoptosis resistance [29]. Numerous clinical studies showed that high KRT7 levels are closely associated with poor prognosis in various types of cancer [20–24, 28–37], and KRT7 has been widely used as a diagnostic and prognostic biomarker in various types of malignant tumor [20–24, 28–37]. In this scenario, the reduction of KRT7 expression, rather than stabilization of KRT7, appears to be a reasonable strategy for tumor suppression. Hence, the role of KRT7-AS in tumorigenesis and apoptosis needs to be clarified.

In light of that the binding of antisense mRNAs to complementary sense mRNAs usually causes degradation of the sense mRNAs in cells [22], we hypothesized that KRT7-AS deficiency may elevate levels of oncogenic KRT7 in cancer cells, thereby increasing tumorigenesis and apoptosis resistance. In the current study, we found that KRT7-AS reduced levels of the oncogenic KRT7 protein in lung and breast cancers; to our surprise, KRT7-AS increased the amounts of phosphatase and tensin homolog deleted on chromosome ten (PTEN), a key tumor suppressor [38–41] and pro-apoptotic modulator in cancer cells [42, 43]. Mechanistically, KRT7-AS directly binds to PTEN protein and protects the protein from degradation in cancer cells, thereby inhibiting tumorigenesis and enhancing apoptosis. KRT7-AS functions as a new tumor suppressor and apoptotic enhancer in cancer cells. Interestingly, we found that there is a novel tumor suppressive RXRα-KRT7-AS-PTEN singling axis in cancer cells, and activation of the RXRα-KRT7-AS-PTEN axis is a novel strategy for developing new therapeutics against cancer.

## Results

### KRT7-AS functions as a new tumor suppressor in lung and breast cancers

**Fig. 1:**
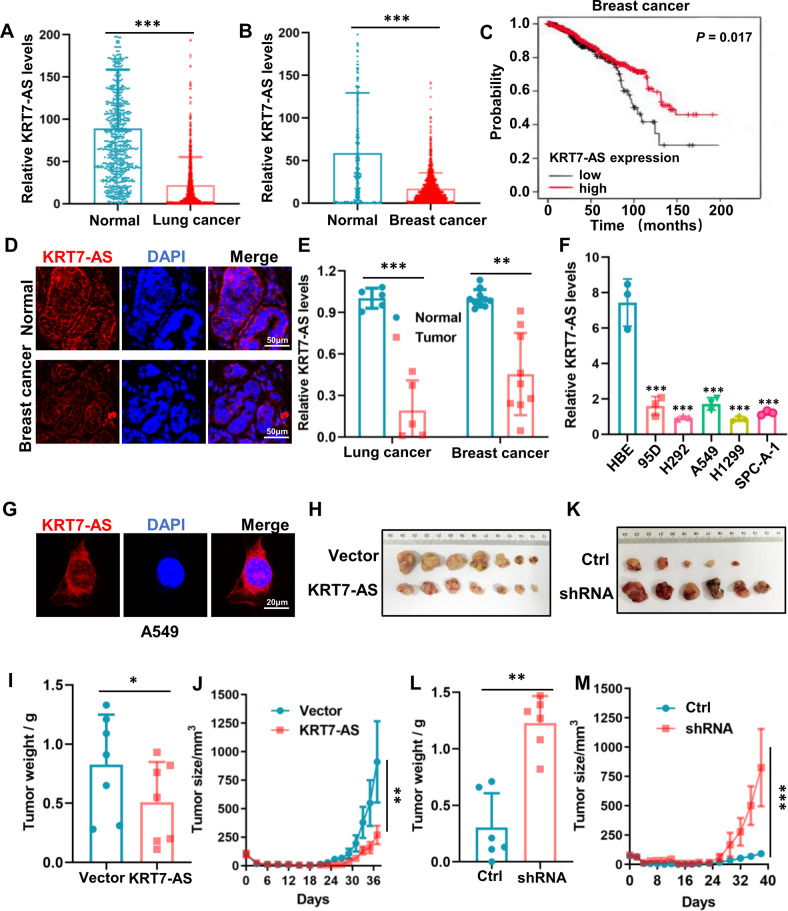
Expression and tumor-suppressive function of the lncRNA KRT7-AS in lung and breast cancers. The levels of KRT7-AS in lung cancer (**A**), breast cancer (**B**), and the adjacent normal tissues were analyzed by integrative analysis using TCGA database. The influence of KRT7-AS levels on the survival of breast cancer patients was also analyzed using TCGA database (**C**). The expression of KRT7-AS (red) in breast cancer and adjacent normal tissues was examined by FISH (**D**), the nuclei were stained with DAPI (blue). The expression levels of KRT7-AS in paired human breast cancer tissues and lung cancer tissues (**E**), and in lung cancer cell lines (95D, NCI-H292, A549, H1299, SPC-A-1), and in the immortalized normal human bronchial epithelial cell line HBE were analyzed by real time qPCR (**F**). The sub-cellular localization of KRT7-AS (red) was examined by FISH (**G**), the nuclei were stained with DAPI (blue). The inhibitory effect of KRT7-AS on tumorigenesis was accessed in xenograft mice (**H**–**J**, *n* = 7). Additionally, the tumor size (**J**) and weight (**I**) of xenograft mice were measured and statistically analyzed, and silencing of KRT7-AS strongly enhanced tumor growth in xenograft mice (**K**–**M**, *n* = 6). The tumor volume was also quantified using GraphPad (**M**). Above data are shown as mean ± SD of three independent replicates. *P < 0.05, **P < 0.01, ***P < 0.001.

We first mined the TCGA database and found that *KRT7-AS* expression levels in nine types of cancer were significantly lower than those in matched normal tissues, including lung cancer, breast cancer (Fig. 1A, B), and other seven malignant tumors (Fig. S1A–G). Particularly, *KRT7-AS* expression levels in 687 samples of lung cancer were reduced 5.3-fold compared with those in adjacent normal lung tissues (*p* < 0.001, Fig. 1A). Additionally, not only was KRT7-AS expression in breast cancer significantly downregulated compared with that in matched normal tissues (*p* < 0.001, Fig. 1B), but also the survival time of patients with low KRT7-AS levels was significantly reduced compared with that of patients with high levels of KRT7-AS (*p* = 0.017, Fig. 1C), suggesting that low KRT7-AS expression is a poor prognostic factor in breast cancer. Conversely, KRT7-AS was moderately upregulated in stomach, colon, and bladder cancers, and notably elevated only in ovarian cancer (Fig. S1H–K).

RNA fluorescence in situ hybridization (FISH) assay showed that the amounts of KRT7-AS in the human breast cancer tissues were lower than those in adjacent normal tissues (Fig. 1D). Real time qPCR indicted that KRT7-AS levels in both types of clinical cancer tissues were significantly lower than those in adjacent normal tissues (Figs. 1E, S2A, B). Consistent with the data from patients, KRT7-AS levels were reduced by threefold to fourfold in five human lung cancer cell lines compared with those in the normal human bronchial epithelial cell line HBE (Fig. 1F). Meanwhile, we used real time qPCR to measure KRT7-AS expression in six breast cancer cell lines, including MDA-MB-231, MCF-7, BT594, MDA-MB-468, MDA-MB-435S, and HS578T. The results showed that KRT7-AS expression levels were downregulated in five out of the six cell lines that we tested (Fig. S2C). FISH assay showed that KRT7-AS was mainly located in the cytoplasm of A549 lung cancer cells (Fig. 1G). Together, these data indicate that KRT7-AS expression is downregulated in lung and breast cancers.

**Fig. 2:**
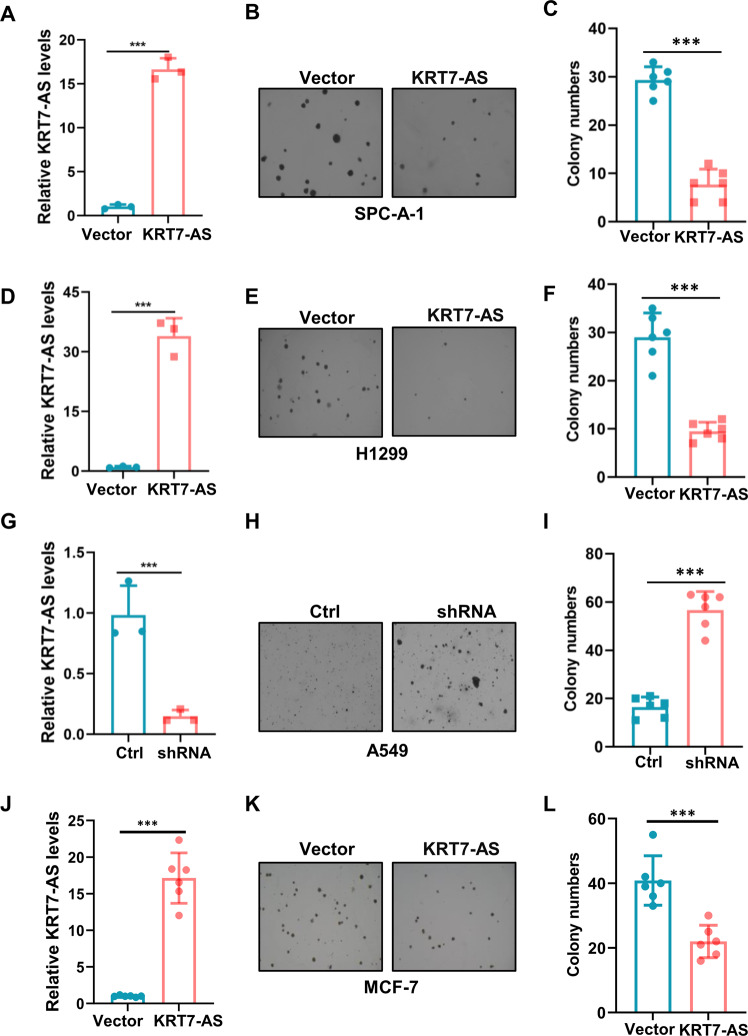
KRT7-AS inhibits lung and breast cancer cell colony formation ability in vitro. KRT7-AS expression levels in lung cancer SPC-A-1 cells were detected by real time qPCR (**A**). Colonies of KRT7-AS-overexpressed SPC-A-1 cells were imaged and quantified (**B**, **C**). The expression levels of KRT7-AS in H1299 were measured by real time qPCR (**D**). The colony formation ability of H1299 cells was accessed (**E**, **F**). The KRT7-AS expression levels in lung cancer A549 cells were detected by real time qPCR (**G**). Colonies of KRT7-AS-silenced A549 cells were imaged and quantified (**H**, **I**). The colony formation ability of breast cancer MCF-7 cells overexpressed KRT7-AS or vector control was accessed (**J**–**L**). Data are shown as mean ± SD of three independent replicates. *P < 0.05, **P < 0.01, ***P < 0.001.

Next, we investigated the impact of KRT7-AS on lung cancer cell tumorigenesis. Colony formation assays showed that the overexpression of KRT7-AS significantly reduced colony numbers in lung cancer SPC-A-1 cells (Fig. 2A–C) and H1299 cells (Fig. 2D–F), whereas silencing of KRT7-AS in lung cancer A549 cells increased the colony numbers (Fig. 2G–I). Meanwhile, KRT7-AS also significantly decreased colony formation ability in breast cancer MCF-7 cells (Fig. 2J–L).

Xenograft mice showed that the tumor weight and volume were significantly smaller in the mice injected with KRT7-AS-overexpressing human lung cancer H1299 cells than in the mice injected with vector control H1299 cells (Figs. 1H–J and S2D). By contrast, the tumor weight and volume were markedly greater in mice injected with KRT7-AS-silenced A549 cells than in mice injected with KRT7-AS-expressing A549 control cells (Figs. 1K–M and S2E). Together, these data suggest that KRT7-AS functions as a new tumor suppressor in breast and lung cancers.

### Upregulation of KRT7-AS expression inhibits cancer cell tumorigenesis and enhances apoptosis

**Fig. 3:**
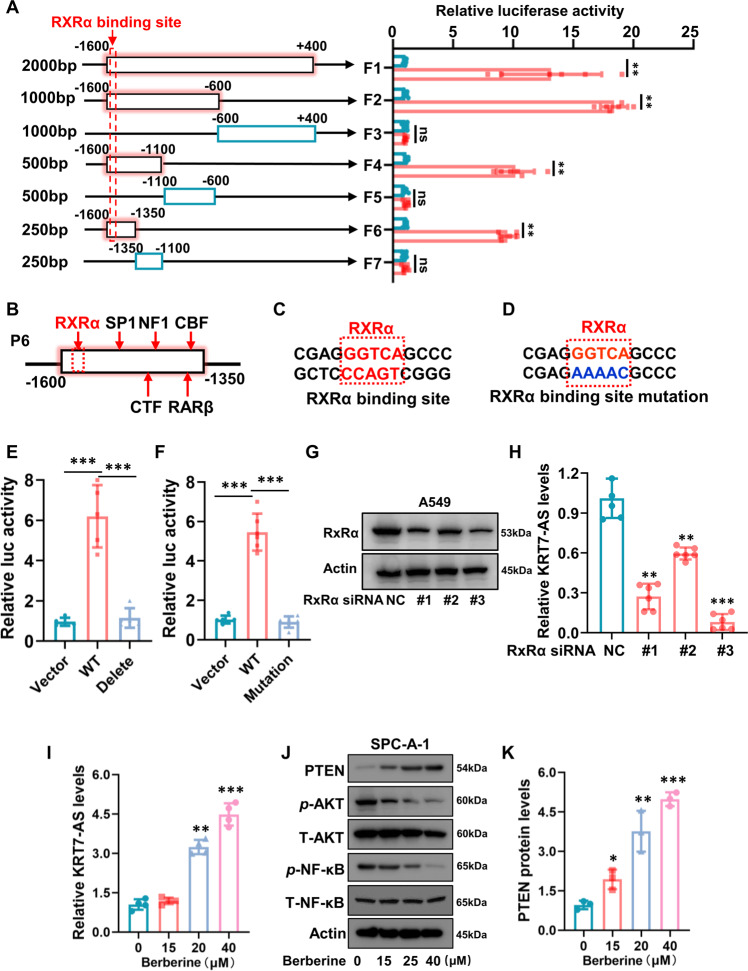
RXRα drives the transcription of KRT7-AS and the RXRα agonist berberine promotes KRT7-AS expression. The *KRT7-AS* genomic DNA fragment (−1600 to +400) from the transcription initial site was separated into seven fragments (**A**, left panel). Luciferase assay showed that the fragments which contain the transcription factor RXRα-binding site had promoter activity (**A**, right panel), and indicated that the promoter DNA from −1600 to −1350 from the transcription initial site was the shortest fragment with strong promoter activity (**A**, **B**). LASAGNA method indicated that the DNA fragment from -1600 to -1350 has six putative transcription factor binding sites (**B**), The core nucleic acids GGTCA in wild type of RXRα-binding site in the sub-fragment P6 (**C**) was mutated to AAAAC (**D**). Luciferase assay showed that both deletion and mutation the core nucleic acids CGAGGGTCAGCCC in the RXRα-binding site lost the promoter activity (**E**, **F**). RXRα levels were detected in RXRα-silenced A549 cells (**G**). KRT7-AS levels were detected in RXRα-silenced A549 cells (**H**). The RXRα agonist berberine significantly increased KRT7-AS transcription levels (**I**), and concurrently raised PTEN protein levels (**J**, **K**). Data are shown as mean ± SD of three independent replicates. *P < 0.05, **P < 0.01, ***P < 0.001.

In light of that KRT7-AS is deficient in various types of cancer and silencing of KRT7-AS promotes tumorigenesis, we hypothesized that upregulation of KRT-AS expression may diminish cancer cell tumorigenesis and enhance apoptosis. In view of that the mechanisms underlying *KRT7-AS* gene transcription have not yet been reported in literature, we first studied the mechanism of *KRT7-AS* transcription in lung cancer cells. Luciferase assay showed that the *KRT7-AS* promoter DNA fragments F2, F4, and F6 had strong promoter activity, whereas F3, F5, and F7 did not have promoter activity (Fig. 3A). The LASAGNA method predicted a common RXRα-binding site with the consensus sequence CGAG**GGTCA**GCCC near the 5′ *KRT7-AS* promoter region in F1, F2, F4, and F6 (Figs. 3A–B, S2F). We deleted or mutated the core nucleic acids **GGTCA** of the RXRα binding site to **AAAAC** in F6-pGL4.17 (Fig. 3C, D). Luciferase assay showed that both deletion and mutation of the GGTCA motif in F6-pGL4.17 completely abolished the *KRT7-AS* promoter activity (Fig. 3E, F), suggesting that the nucleic acids GGTCA are the core consensus sequence in the *KRT7-AS* promoter responsible for *KRT7-AS* transcription, which implies that RXRα is likely the key transcription factor to drive *KRT7-AS* transcription. Then we downregulated RXRα using three different RXRα siRNAs (Fig. 3G). Real time qPCR revealed that RXRα siRNA#3 markedly inhibited KRT7-AS expression in lung cancer A549 cells (Fig. 3H). These data confirm that the transcription factor RXRα controls expression of KRT7-AS. On the other hand, we explored whether activation of RXRα affects KRT7-AS expression and found that the RXRα agonist berberine [44, 45] significantly increased the levels of KRT7-AS in SPC-A-1 cells (Fig. 3I). These data indicate that RXRα triggers *KRT7-AS* transcription in lung cancer cells.

To our surprise, KRT7-AS raised levels of the tumor suppressor PTEN protein, while berberine also increased PTEN protein amounts in cancer cells (Fig. 3J, K). Additionally, KRT7-AS suppressed the phosphorylation of AKT and NF-κB, while berberine diminished phosphorylated AKT and NF-κB in a concentration-dependent manner (Figs. 3J and S2G, H). These data suggest that KRT7-AS is a new PTEN enhancer, and berberine is a novel KRT7-AS inducer.

**Fig. 4:**
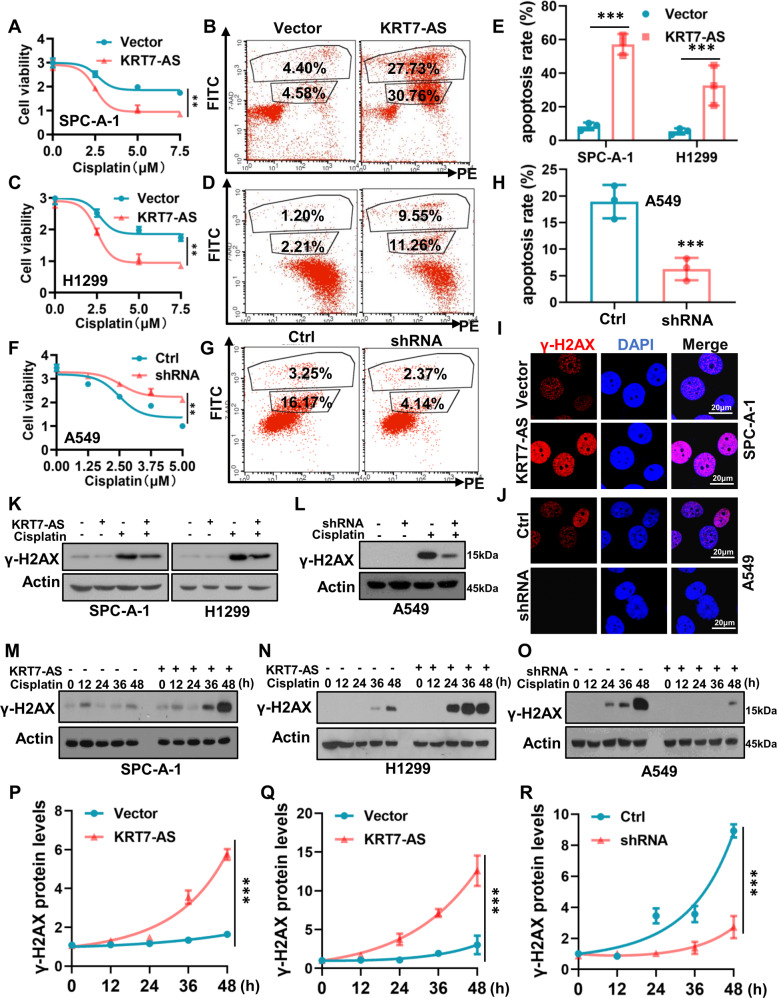
Upregulation of KRT7-AS enhances cisplatin-induced DNA damage and increases lung cancer cell sensibility to the drug. Cell Titer-Glo luminescent (CTG) assay showed that KRT7-AS overexpression enhanced the sensibility of SPC-A-1 (**A**) and H1299 (**C**) lung cancer cells to the anti-cancer drug cisplatin. Flow cytometry analysis showed that KRT7-AS overexpression significantly enhanced cisplatin-induced apoptosis of SPC-A-1 (**B**, **E**) and H1299 (**D**, **E**) lung cancer cells. CTG and flow cytometry analysis showed that in KRT7-AS- silenced A549 lung cancer cells, reduction of KRT7-AS levels diminished cisplatin-induced apoptosis (**F**, **G**, **H**). The effect of KRT7-AS on the DNA damage marker γ-H2AX in KRT7-AS-overexpressed SPC-A-1 and KRT7-AS-silenced A549 cells in the presence of cisplatin was accessed by IF staining (**I**, **J**) and Western blotting (**K**, **L**). Time course study showed that KRT7-AS-induced elevation of γ-H2AX levels in SPC-A-1 (**M**, **P**), H1299 (**N**, **Q**), and A549 (**O**, **R**) lung cancer cells was in a time-dependent manner (M-R). Data are shown as mean ± SD of three independent replicates. **P < 0.01, ***P < 0.001.

Then, we investigated whether KRT7-AS influences the sensitivity of lung cancer cells to the anti-cancer drug cisplatin and regulates apoptosis. The results showed that KRT7-AS overexpression significantly increased the sensitivity of lung cancer cells to cisplatin (Fig. 4A, C), whereas silencing of KRT7-AS had the opposite effect (Fig. 4F). Additionally, KRT7-AS enhanced cell sensibility to cisplatin in breast cancer MCF-7 cells (Fig. S2I). Strikingly, KRT7-AS overexpression markedly enhanced cisplatin-induced apoptosis of lung cancer SPC-A-1 and H1299 cells by more than fivefold (Fig. 4B, D, E), whereas silencing of KRT7-AS in A549 cells significantly decreased cisplatin-induced apoptosis (Fig. 4G, H), suggesting that KRT7-AS enhances cancer cell apoptosis.

Next, we investigated the mechanisms underlying KRT7-AS induced lung cancer cell apoptosis. The immunofluorescent staining indicated that KRT7-AS-overexpressing tumor cells were highly susceptible to cisplatin, as indicated by high levels of γ-H2AX, a classic marker of DNA strand breaks (Fig. 4I), conversely, KRT7-AS-silenced lung cancer A549 cells showed a decrease in γ-H2AX levels (Fig. 4J). Additionally, Western blotting showed that KRT7-AS overexpression elevated the levels of cisplatin-induced γ-H2AX in lung cancer cells (Fig. 4K), whereas silencing of KRT7-AS decreased the amount of cisplatin-induced γ-H2AX in A549 cells (Fig. 4L). Furthermore, KRT7-AS induced elevation of γ-H2AX levels in lung cancer cells was in a time-dependent manner (Fig. 4M, N, P, Q); by contrast, silencing of KRT7-AS notably reduced the levels of γ-H2AX (Fig. 4O, R).

**Fig. 5:**
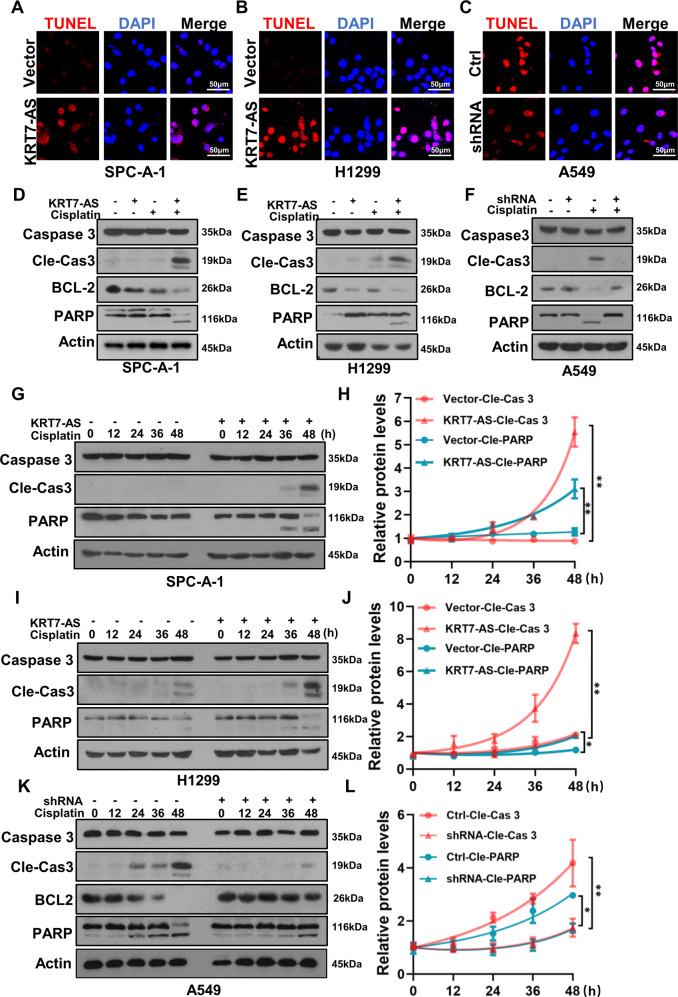
KRT7-AS enhances cisplatin-induced apoptosis and activates apoptotic signaling pathways in cancer cells. TUNEL assay showed that KRT7-AS enhanced cisplatin-induced apoptosis in SPC-A-1 (**A**) and H1299 (**B**) lung cancer cells, whereas, silencing of KRT7-AS in A549 (**C**) lung cancer cells reduced apoptosis. The levels of Caspase 3, cleaved-Caspase 3, BCL-2, and PARP in KRT7-AS-overexpressed SPC-A-1 (**D**) and H1299 (**E**) lung cancer cells, or KRT7-AS-silenced A549 (**F**) lung cancer cells were measured by Western blotting. Time course study showed that KRT7-AS elevated the levels of cleaved-Caspase3 and cleaved PARP in cisplatin concentration- and time-dependent manner (**G**–**L**). Data are shown as mean ± SD of three independent replicates. *P < 0.05, **P < 0.01.

The TUNEL assay showed that overexpression of KRT7-AS promoted apoptosis of SPC-A-1 and H1299 lung cancer cells (Fig. 5A, B), while silencing of KRT7-AS diminished apoptosis of the cells (Fig. 5C). Mechanically, overexpression of KRT7-AS significantly increased levels of cleaved-caspase 3 and cleaved-PARP in lung cancer cells in the presence of cisplatin (Fig. 5D, E); whereas silencing of KRT7-AS significantly reduced levels of cleaved-caspase 3 and cleaved-PARP in A549 cells (Fig. 5F). Either elevation of cleaved-caspase3 and cleaved-PARP levels by KRT7-AS overexpression (Fig. 5G–J) or reduction of cleaved-caspase3 and cleaved-PARP levels in cancer cells by silencing of KRT7-AS (Fig. 5K, L) was in a time-dependent manner. Together, these data indicate that KRT7-AS enhances cancer cell apoptosis and is a new apoptosis enhancer.

### The novel RXRα-KRT7-AS-PTEN signaling axis inhibits tumorigenesis and enhances apoptosis in cancer cells

**Fig. 6:**
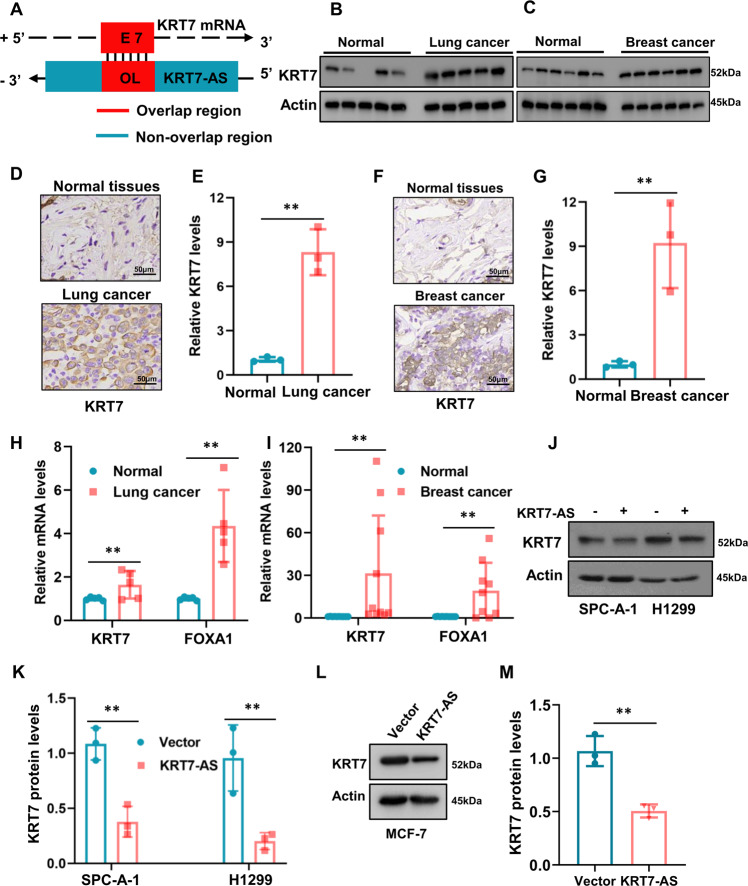
KRT7-AS increases the levels of oncogenic KRT7 in lung cancer and breast cancer. The relationship between KRT7 and KRT7-AS in genomic DNA is depictured in (**A**). KRT7 protein levels in lung cancer (**B**) and breast cancer (**C**) were detected by Western blotting. The levels of KRT7 protein in the lung cancer (**D**, **E**) and breast cancer (**F**, **G**) tissues and in the paired normal tissues were examined by IHC. Expression levels of KRT7 downstream *FOXA1* gene in lung cancer (**H**) and breast cancer (**I**) were detected using real time qPCR. Western blotting showed that KRT7 protein was decreased in KRT7-AS-overexpressed SPC-A-1 and H1299 lung cancer cells (**J**, **K**). In KRT7-ASoverexpressed MCF-7 cells, KRT7 protein levels were reduced (**L**, **M**). Data are shown as mean ± SD of three independent replicates. **P < 0.01.

Next, we set to study the mechanisms underlying KRT7-AS-meditated tumor suppression and pro-apoptosis. In light of that oncogenic KRT7 promotes carcinogenesis [20–24, 28–37] and the binding of antisense mRNAs to complementary sense mRNAs usually causes degradation of the sense mRNAs in cells [22], we first investigated whether KRT7-AS reduces KRT7 levels in cancers. The alignment between the KRT7-AS and KRT7 mRNAs revealed an overlapping region of 213 nucleic acids with 100% nucleotide complementarity (Fig. 6A). Next, we examined whether KRT7-AS deficiency in lung and breast cancers was related to the high levels of oncogenic KRT7 in clinical cancer patients. Western blots showed that KRT7 levels were significantly higher in the lung and breast tumor tissues than that in the adjacent normal tissues (Fig. 6B, C). Immunohistochemical analysis confirmed that KRT7 was overexpressed in the clinical lung cancer tissues (Fig. 6D, E) and breast cancer tissues (Fig. 6F, G), of note, KRT7-AS levels were notably reduced in lung and breast cancer tumor tissues as stated before (Fig. 1A, B). Strikingly, qPCR showed that KRT7 mRNA levels were increased by sevenfold and ninefold in the lung and breast tumors, respectively; meanwhile, the KRT7 downstream oncogenic forkhead box protein A1 (FOXA1) [46, 47] was markedly elevated (Fig. 6H, I). Convincingly, Western blots showed that overexpression of KRT7-AS significantly reduced KRT7 levels in lung cancer SPC-A-1 and H1299 cells (Fig. 6J, K), and breast cancer MCF-7 cells (Fig. 6L, M). In short, these data imply that KRT7-AS reduces expression of oncogenic KRT7 and downstream tumorigenic FOXA1 in cancer tissues and cells, implying that inhibition of oncogenic KRT7 and FOXA1 is one of the mechanisms underlying KRT7-AS-mediated tumor suppression and pro-apoptosis.

**Fig. 7:**
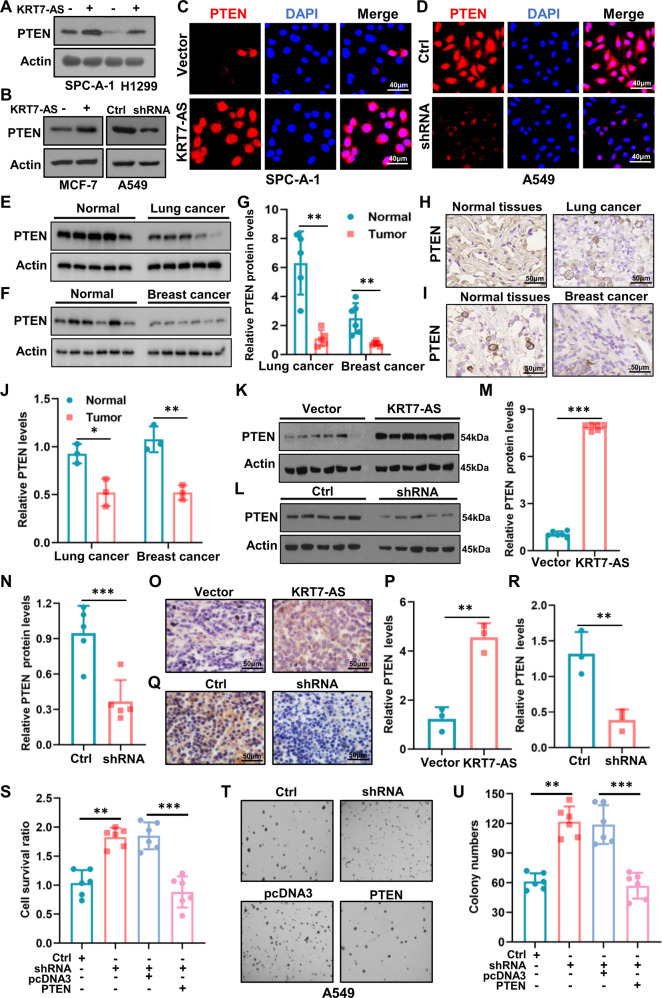
KRT7-AS elevates amounts of the key tumor suppressor PTEN in cancer cells and inhibits tumorigenesis. Western blotting showed that overexpression of KRT7-AS elevated PTEN protein levels in SPC-A-1 and H1299 lung cancer cells (**A**), and in MCF-7 breast cancer cells (**B**), whereas silencing of KRT7-AS reduced the levels of PTEN in A549 lung cancer cells (**B**). The effect of either overexpression of KRT7-AS or silencing of KRT7-AS on PTEN levels in SPC-A-1 cells (**C**) and A549 lung cancer cells (**D**) was accessed by IF staining. Expression of PTEN in lung cancer (**E**, **G**) and breast cancer tissues (**F**, **G**), and their paired normal tissues (**E**–**G**) was detected by Western blotting. PTEN protein levels in lung cancer (**H**, **J**) and breast cancer (**I**, **J**) tissues were measured by IHC staining. The effect of KRT7-AS on PTEN levels in the tumor tissues from the xenograft mice was accessed by western blotting (**K**–**N**) and IHC staining (**O**–**R**), respectively. Cell viability and colony formation were detected in KRT7-AS-silenced or PTEN re-overexpressed A549 cells, and control cells (**S**–**U**). Data are shown as mean ± SD of three independent replicates. **P < 0.01, ***P < 0.001.

To explore other mechanisms underlying KRT7-AS-mediated anti-tumorigenesis, we performed RNA-Seq and gene expression profile analysis. The results showed that there are 85 differentially expressed RNAs in KRT7-AS-overexpressing lung cancer cells as compared with control lung cancer cells (fold change > 3, *p* < 0.05; Fig. S3), and sixteen signaling pathways were obviously changed in the KRT7-AS-overexpressing lung cancer cells (Fig. S4). As stated before, berberine promotes expression of KRT7-AS (Fig. 3I) and increases PTEN protein levels (Fig. 3J, K). Accordingly, we set to clarify the relationship between KRT-AS and PTEN. Western blots showed that KRT7-AS overexpression elevated PTEN protein levels in SPC-A-1 cells and H1299 cells (Fig. 7A), and MCF-7 cells; conversely, silencing of KRT7-AS using shRNA in A549 cells markedly reduced PTEN levels (Fig. 7B). Immunofluorescence analysis indicated that overexpression of KRT7-AS markedly increased PTEN protein levels in SPC-A-1 cells (Fig. 7C), whereas silencing of KRT7-AS notably decreased PTEN protein levels in A549 cells (Fig. 7D); of note, the PTEN mRNA levels were not affected by either overexpression of KRT7-AS or silencing of KRT7-AS in the cancer cells (Fig. S5B, C, E). Consistent with the results, PTEN protein levels were significantly reduced in the human cancer tissues compared with the adjacent normal tissues (Fig. 7E–J). Convincingly, overexpression of KRT7-AS increased PTEN protein levels in the tumors of the xenograft mice (Figs. 7K, M, O, P; S5J), whereas silencing of KRT7-AS resulted in a decrease in PTEN protein levels in the tumors (Fig. 7L, N, Q, R; S5K), and qPCR showed that there was no difference in PTEN mRNA levels between the two groups of xenograft mice (Fig. S5F–I). Together, these results imply that KRT7-AS increases PTEN protein levels in cancer tissues and cells likely by post-transcriptionally modifying PTEN.

Furthermore, silencing of KRT7-AS significantly increased the viability of the A549 cells, convincingly, re-overexpression of PTEN in the KRT7-AS-silenced A549 cells significantly reduced cell sensibility to cisplatin and colony formation of the cells (Figs. 7S–U, S6A–C). Silencing of KRT7-AS by shRNA increased levels of phosphorylated *p*-AKT and *p*-NF-κB, which was reversed by both overexpression of PTEN (Fig. S6D–G) and PTEN inhibitor (Fig. S6H, I). Together, these data suggest that PTEN is involved in KRT7-AS-mediated tumor suppression and pro-apoptosis, and PTEN is at the downstream of KRT7-AS.

**Fig. 8:**
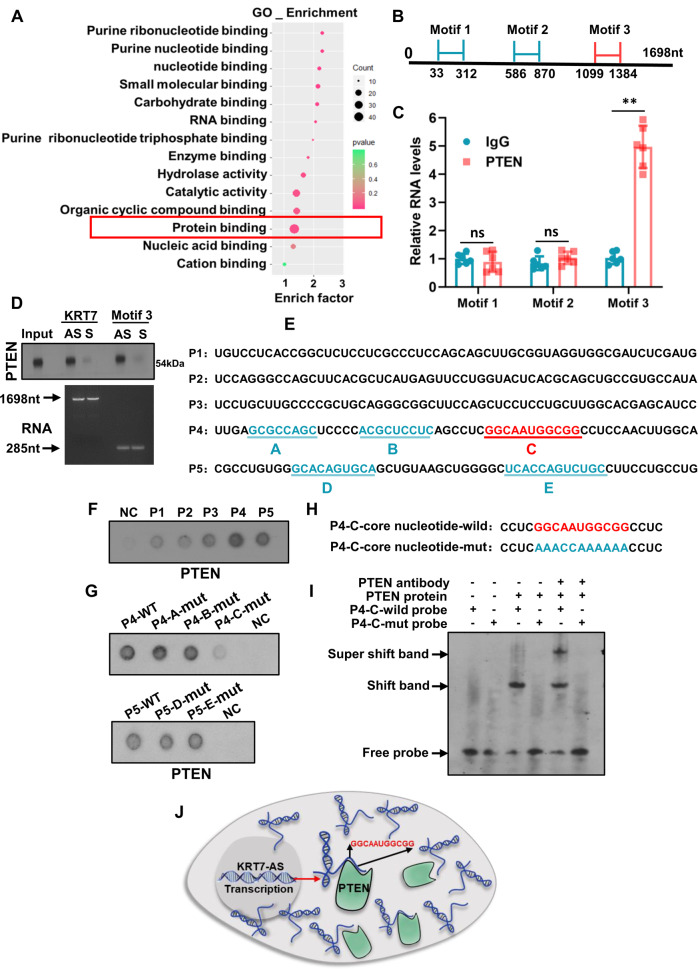
KRT7-AS directly binds to PTEN and stabilizes the protein in lung cancer cells. GO analysis indicated that KRT7-AS possesses putative protein-binding motifs with the highest count as shown in a red frame (**A**). CatRAPID analysis showed that KRT7-AS has three putative motifs that potentially bind to PTEN protein (**B**). RIP assay showed that the motif 3 of KRT7-AS bound to PTEN protein (**C**), RNA pulldown assay indicated that the anti-sense KRT7-AS (full length or motif 3), but not the sense KRT7-AS (full length or motif 3), bound to PTEN protein directly (**D**). CatRAPID further predicted that there are five sub-fragments (**A**–**E**) in the motif 3 (**E**). Dot blot showed that the binding of fragments P4 and P5 with PTEN protein was stronger than the other 3 fragments (**F**). Dot blot showed that the P4 mutation (P4-C-mut) lost its ability for binding to PTEN protein (**G**, **H**). EMSA assay showed that wild type of the P4-C sub-fragment GGCAAUGGCGG bound to PTEN protein directly, whereas mutant P4-C lost the binding capability (**I**). Schematic figure showed the binding of KRT7-AS to PTEN protein (**J**). Data are shown as mean ± SD of three independent replicates. **P < 0.01.

Next, we explored whether KRT7-AS directly binds to the PTEN protein. The GO enrichment analysis showed that KRT7-AS has potential protein-binding capability (Fig. 8A). CatRAPID software predicted that there are three putative motifs in KRT7-AS that putatively bind to the PTEN protein with high scores, each motif is 285 nucleotides in length, and we refer to them as motifs 1, 2, and 3, respectively (Fig. 8B). RNA immunoprecipitation assay indicated that the motif 3, rather than motifs 1 and 2, bound to the PTEN protein in lung cancer cells, but the sense KRT7 and sense motif 3 mRNAs did not bind to PTEN protein (Fig. 8C). RNA pulldown assays also confirmed direct binding of the full length of KRT7-AS (1698 bp) and motif 3 (285 bp) mRNAs to the PTEN protein (Fig. 8D).

Then, we divided the KRT7-AS motif 3 mRNA into five small fragments (57nt each) named P1, P2, P3, P4, and P5 (Fig. 8E). We found that the binding of P4 and P5 was much stronger than that of the fragments P1, P2, and P3 (Fig. 8F). P4 and P5 possess three and two motifs that putatively bind to PTEN, respectively. We named these motifs P4-A, P4-B, P4-C, P5-D, and P5-E (Fig. 8E). Mutation of P4-C motif almost completely lost its ability to bind PTEN. By contrast, Mutations of P4-A, P4-B, P5-D, and P5-E motifs could still bind PTEN (Fig. 8G), suggesting that the P4-C motif is responsible for binding to the PTEN protein (Fig. 8H). Furthermore, gel mobility shift assay and super shift assay showed that the wild-type P4-C motif (GGCAAUGGCGG) bound the PTEN protein directly, but the mutant P4-C motif could not bind to PTEN (Fig. 8I). These data indicate that the KRT7-AS P4-C motif GGCAAUGGCGG directly binds and stabilizes the PTEN protein (Fig. 8J).

**Fig. 9:**
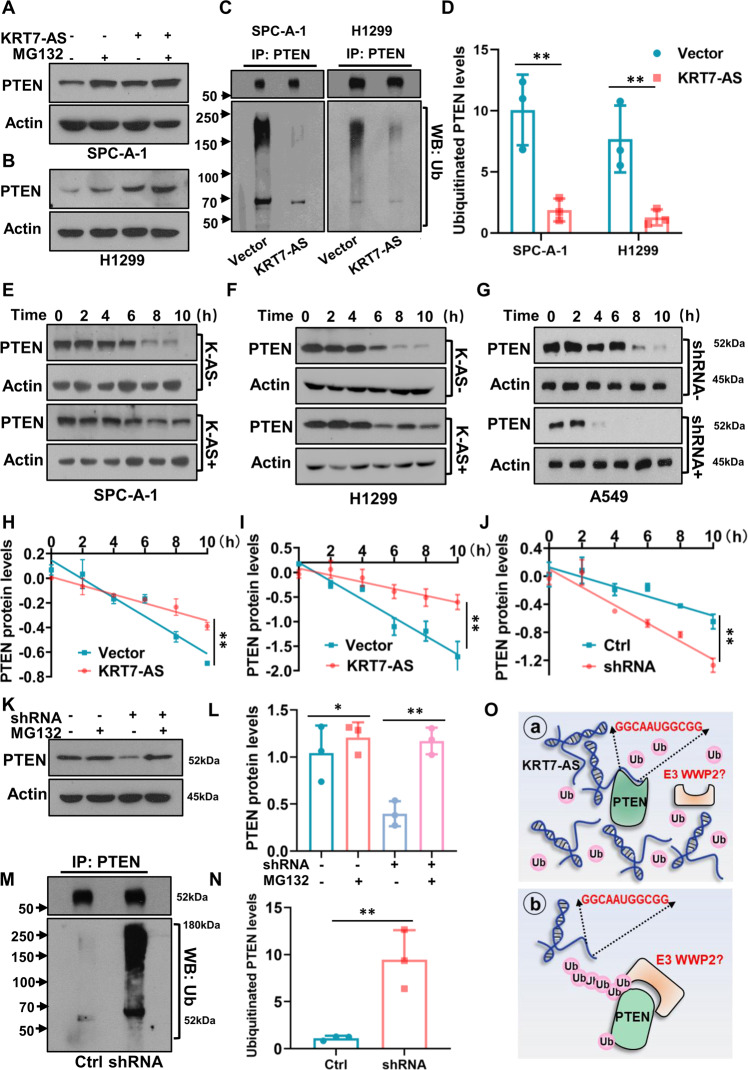
KRT7-AS protects PTEN protein from degradation via inhibiting the ubiquitination-proteasome system in lung cancer cells. The ubiquitination inhibitor MG132 raised PTEN levels in SPC-A-1 (**A**) and H1299 (**B**) lung cancer cells. KRT7-AS overexpression significantly reversed the ubiquitination of PTEN protein in SPC-A-1 and H1299 cells (**C**, **D**). The half-life of PTEN protein was prolonged by KRT7-AS overexpression in the presence of the protein synthesis inhibitor CHX in SPC-A-1 (**E**, **H**) and H1299 cells (**F**, **I**). Silencing of KRT7-AS shortened the half-life of PTEN protein in A549 lung cancer cells (**G**, **J**). KRT7-AS-mediated degradation of PTEN protein in A549 cells was significantly reversed by the ubiquitination inhibitor MG132 (**K**, **L**). Silencing of KRT7-AS significantly increased ubiquitination of PTEN protein in A549 cells (**M**, **N**). Schematic figure showed that KRT7-AS binds to PTEN and protects the protein from degradation by ubiquitination system (**O**). Data are shown as mean ± SD of three independent replicates. *P < 0.05, **P < 0.01.

In light of that PTEN plays a key role in tumor suppression and pro-apoptosis [38–41], we set to elucidate the mechanisms underlying KRT7-AS-mediated PTEN protein stabilization, anti-tumor, and pro-apoptosis. We used the ubiquitination-proteasome inhibitor MG132 to treat lung cancer cells. The results showed that MG132 increased PTEN protein levels in SPC-A-1 and H1299 cells (Fig. 9A, B), and overexpression of KRT7-AS further elevated PTEN protein levels in those cells (Fig. 9A, B). Co-immunoprecipitation showed that the levels of ubiquitinated PTEN protein were markedly reduced in KRT7-AS-overexpressing lung cancer cells compared with those in control cells (Fig. 9C, D). Additionally, PTEN protein decay was reduced by KRT7-AS overexpression in SPC-A-1 and H1299 cells (Fig. 9E, F). Analysis of protein dynamics indicated that KRT7-AS overexpression prolonged the half-life of PTEN in both SPC-A-1 (Fig. 9H) and H1299 cells (Fig. 9I), whereas the half-life of PTEN was significantly shortened in KRT7-AS-silenced A549 cells compared with that in control cells (Fig. 9G, J). Furthermore, the PTEN protein levels in KRT7-AS-silenced A549 cells were elevated in the presence of ubiquitination inhibitor MG132 (Fig. 9K, L). Immunoprecipitation analysis revealed that the amount of ubiquitinated PTEN was notably increased in KRT7-AS-silenced A549 cells (Fig. 9M, N). Collectively, these data indicate that KRT7-AS stabilizes PTEN protein in cancer cells by protecting the protein from degradation by the ubiquitination-proteasome system (Fig. 9O).

## Discussion

Activation of various oncogenes and concurrent defects in endogenous tumor suppressors and pro-apoptotic genes play pivotal roles in uncontrolled tumor growth and apoptosis resistance [8–16, 38–43]. How to effectively suppress oncogenes and simultaneously activate tumor suppressors and pro-apoptotic genes is a big challenge in cancer research field. KRT7-AS is deficient in nine types of cancers; however, the effect of KRT7-AS deficiency on carcinogenesis remains enigmatic. In this study, we found that KRT7-AS functions as a new tumor suppressor and apoptotic enhancer in lung and breast cancers through reduction of oncogenic KRT7 levels and stabilization of PTEN protein. We also found that there is a novel tumor suppressive RXRα-KRT7-AS-PTEN signaling axis in cancer, and activation of the signaling axis by RXRα agonist berberine inhibits tumorigenesis and enhances apoptosis. Our findings provide new insight into lncRNA-mediated tumor suppression and pro-apoptosis, and offer a new platform for the development of novel therapeutics against cancer.

It has been reported that KRT7-AS enhances tumorigenesis in gastric cancer [17] and colorectal cancer [18] by stabilizing sense KRT7 mRNA. This interpretation may be farfetched for majority of cancers, however, from the standpoint of molecular biology, cancer biology, and clinical evidence, because the binding of antisense mRNA to complementary sense mRNA usually causes either degradation or dysfunctional translation of the latter. It is well established that KRT7 is oncogenic [24–28], and its overexpression promotes tumorigenesis and is closely associated with poor prognosis in various types of cancer [20–24, 28–37]. Accordingly, KRT7 has been widely used as a biomarker for the diagnosis and prognosis of various cancers [20–23, 28–30, 37]. Hence, the impact of KRT7-AS on tumorigenesis and apoptosis needs to be verified. In the current study, we found that KRT7-AS is deficient in lung and breast cancers, low expression of KRT7-AS is associated with poor prognosis in breast cancer patients. Additionally, we found that silencing of KRT7-AS notably increases KRT7 levels and enhances tumorigenic and anti-apoptotic effects in cancer cells; while overexpression of KRT7-AS reduces levels of oncogenic KRT7 in lung and breast cancer tissues and tumor cells, resulting in tumor suppression and pro-apoptosis effects. Thus, KRT7 mRNA de-stabilization, rather than KRT7 mRNA stabilization, in cancer cells should be applied in tumor suppression and pro-apoptosis. Accordingly, we propose that upregulation of tumor-suppressive KRT7-AS in cancer cells is a sensible strategy for developing novel therapeutics against lung and breast cancers, and other KRT7-AS deficient malignant tumors as well.

The tumor suppressor PTEN plays a pivotal role in the prevention of cancer initiation and progression [48], and pro-apoptosis [38–41]. In general, PTEN is deficient in various types of cancer because it is either downregulated or de-stabilized by ubiquitin-mediated proteasomal degradation in approximate 90% cancer patients [49–52]. How to prevent PTEN from degradation and elevate PTEN levels in cancer cells therefore a scientific problem to be resolved. In this study, we found that KRT7-AS binds to PTEN protein directly through the core nucleic acids GGCAAUGGCGG. This protects the protein from degradation by the ubiquitination-proteasome system, consequently elevating PTEN protein levels in cancer cells. On the other hand, berberine-induced overexpression of KRT7-AS also stabilizes PTEN and increases PTEN levels in tumor cells. Thus, KRT7-AS acts as “one stone hits two birds”, that concurrent reduction of oncogenic KRT7 levels and elevation of tumor-suppressive PTEN amounts in cancer cells.

There is a limitation in the current study, the molecular mechanism underlying KRT7-AS-mediated prevention of PTEN protein from degradation by ubiquitination-proteasome system needs to be further elucidated. In various types of cancer, PTEN protein levels are downregulated mainly via ubiquitination and degradation. It has been reported that the E3 ligases NEDD4 [50, 52] and WWP2 [53, 54] are involved in ubiquitination and degradation of PTEN protein. In this study, we found that KRT7-AS inhibits PTEN ubiquitination and degradation; whereas, which E3 ligase is responsible for KRT7-AS- mediated inhibition of PTEN ubiquitination remains to be investigated. We utilized catRAPID software to analyze the potential binding between KRT7-AS and E3 ligases. The results indicated that KRT7-AS might interact with the E3 ligase WWP2, and KRT7-AS might bind to PTEN AA101-152 (containing a core phosphatase functional domain) [55] and AA303-350 (containing a C2 domain) [56], respectively. In light of that WWP2 binds to the C2 domain of PTEN, thereby causing PTEN ubiquitination and degradation, we speculate that the binding of KRT7-AS to the C2 domain in PTEN sterically blocks the binding of WWP2 to PTEN protein, therefore preventing PTEN protein from ubiquitination and degradation.

The anti-cancer drug cisplatin causes DNA damage and cancer cell apoptosis, and is widely used to treat various types of cancer, however, most tumors readily develop cisplatin resistance [57, 58], and cancer cells strongly activate DNA repair machinery to heal DNA strand breaks, resulting in drug resistance, apoptosis resistance, and tumor progression [59–65]. Finding a way to effectively inhibit DNA repair and simultaneously reduce drug and apoptosis resistance is a major task in cancer research field. We found that overexpression of KRT7-AS increases sensitivity of cancer cells to cisplatin and concurrently inhibits DNA repair and enhances apoptosis in cancer cells, thus diminishing drug and apoptosis resistance, resulting in effective tumor suppression. Our findings provide a new strategy for cancer therapy.

The mechanism underlying transcriptional regulation of *KRT7-AS* is unclear. We found that the core nucleic acids GGTCA in the *KRT7-AS* promoter region are critical for transcription of *KRT7-AS*, and transcription factor RXRα [66] drives *KRT7-AS* transcription. The RXRα agonist berberine promotes KRT7-AS expression and increases PTEN levels, activates the tumor suppressive RXRα-KRT7-AS-PTEN axis, inhibits tumorigenic AKT and NF-κB signaling pathways, tumorigenesis, and promotes apoptosis. Thus, KRT7-AS and berberine are potential cancer candidates for the development of novel therapeutics against cancers.

In summary, KRT7-AS reduces cancer cell tumorigenesis, inhibits tumor growth, and enhances cancer cell apoptosis mainly through decreasing oncogenic KRT7 expression and increasing tumor-suppressive PTEN protein levels in cancer cells. KRT7-AS functions as a new tumor suppressor and apoptotic enhancer in lung and breast cancers. There is a novel tumor suppressive RXRα-KRT7-AS-PTEN signaling axis in cancer cells. Therefore, upregulation of KRT7-AS expression and activation of the RXRα-KRT7-AS-PTEN signaling axis are novel strategy for cancer therapy (Graphic summary).

## Materials and methods

### Materials

Monoclonal antibody against β-actin and various chemicals were from Sigma-Aldrich (St. Louis, MO). TaqTM DNA Polymerase was from TaKaRa Biotechnology Co. Ltd (6119, Shiga, Japan). Revert Aid TM First Strand cDNA Synthesis Kit was from Vazyme (#R312-01, Nanjing, China). Antibodies against PTEN (#D4.3), rabbit monoclonal antibody to phosphorylated AKT473 (#587F11), total AKT (#11E7), total NF-κB (#D141E2), phosphorylated NF-κB (#93H1), Caspase 3 (#9662), cleaved-caspase3 (#5A1E), γ-H2AX (#D17A3), PARP (#46D11) and BCL-2 (#D55G8) were obtained from Cell Signaling Technology (Boston, USA). FISH kit (#C10910) was from RiboBio (Shanghai, China). Secondary antibodies were obtained from Thermo Scientific (#A27041, #31320, Waltham, USA). Human lung cancer cell lines SPC-A-1, H1299, NCI-H292, 95D, and A549 were from ATCC (Manassas, Virginia).

### Cell culture

The human lung cancer cell lines SPC-A-1, H1299, NCI-H292, 95D, and A549 were cultured in DMEM supplemented with 10% fetal bovine serum (the complete medium), at 37 °C in a humidified atmosphere of 5% CO_2_ as previously described [67].

### Cell proliferation assay

SPC-A-1, A549, and H1299 cells were seeded in a 96-well plate and incubated with cisplatin at concentrations of 0–30 μM. After three days of treatment, 10 μl of the Cell Titer-Glo (CTG) luminescent solution (5 mg/ml) was added to each well and incubated for 10 min at 37 °C, then the dual fluorescence wavelength absorbance at 490 nm was measured using SpectraMax M5 multi-detection reader.

### Colony formation assay

The 35 mm dishes were coated with 1 ml 0.5% agarose supplemented with the complete medium. After the bottom layer solidified, 5000 either SPC-A-1 or H1299, or A549 cells in 1 mL of the complete medium were first mixed with 0.15 mL 4% low melting agarose, and then put onto the dishes and let it to solidify. The dishes were incubated at 37 °C in 5% CO_2_ for 14 days, the colonies were counted and imaged under a Zoom-Stereo microscope SZX16 (OLYMPUS, Tokyo, Japan).

### Stable transfection of cell lines

Either KRT7-AS cDNA or KRT7-AS shRNA, or normal control shRNA was cloned into the lentivirus vector GV112-IRES-GFP. The 293T cells were transfected with these constructs to produce infectious viruses. The lung cancer cell lines SPC-A-1, H1299, and A549 were infected with the infectious viruses, and the transfected cells were selected using puromycin. The expression of KRT7-AS in these stably transfected cells was measured using RT-PCR and real time qPCR, respectively.

### Tumor xenograft in mice

Tumor xenograft in mice was conducted in accordance with protocols approved by the Institutional Animal Care and Use Committee (IACUC) of Soochow University as previously described [68], and we confirm that all experiments conform to all relevant regulatory standards, and the mice were randomly divided into two groups. In brief, 10^7^ human lung cancer H1299 or A549 cells, which were either overexpression or silencing of KRT7-AS, were subcutaneously injected in 8-week-old female nude mice, respectively. Tumor volume was measured and calculated according to the formula: tumor volume = 0.55 × length × width^2^.

### RT-PCR and quantitative real-time PCR

Total RNA was extracted from A549, H1299, and SPC-A-1 cells with different treatment. The RT-PCR and quantitative real time PCR (qPCR) were conducted as we described before [68].

### Western blotting

Proteins from SPC-A-1, H1299 and A549 cells were extracted using the MPER Mammalian Protein Extraction Kit, and Western blotting was performed as previously described [68]. The proteins were resolved by sodium dodecyl sulfate polyacrylamide gel electrophoresis (SDS-PAGE) with Tris–glycine running buffer and transferred to nitrocellulose membranes. The membranes were blocked with 5% nonfat milk and incubated with primary antibodies at 4 °C overnight, followed by incubation with HRP-coupled secondary antibody for 1 h at 22 °C and the enhanced chemiluminescence detection reagents, and exposed to X-ray film.

### Co-immunoprecipitation

Co-immunoprecipitation (Co-IP) was carried out as previously described [69]. Four hundred microliters of the A549 cell lysates were incubated with primary monoclonal antibody (1:500) or mouse normal IgG as a control at 4 °C for 4 h, then further incubated with 20 μl of 1% fetal bovine serum (BAS)-blocked protein A/G agarose beads at 4 °C overnight with rotation. The immune complexes were released from the beads in SDS loading buffer. The proteins were detected by Western blotting as mentioned above.

### Immunofluorescence microscopy

SPC-A-1, H1299, and A549 cells were seeded onto glass cover slips for 48 h, then fixed with 4% paraformaldehyde, blocked with 1% BSA for 1 h, and incubated with primary antibodies at 4 °C overnight. Then the cells were incubated with secondary antibodies for 1 h at room temperature, and counterstained with 4,6-diamidino-2-phenylindole (DAPI). The slides were observed using confocal microscopy. In addition, the tumor tissues from xenograft mice were sectioned, deparaffinized, blocked with 5% BSA, and incubated with primary antibody against PTEN, followed by the addition of secondary antibodies, counterstained with DAPI, and subjected to be imaged under confocal microscopy.

### Immunohistochemical staining

The tumor tissues from the xenograft mice were sectioned, deparaffinized, and blocked with 5% BSA; then the slides were stained with anti-PTEN primary antibody at 4 °C overnight, followed by the incubation with goat anti-mouse/rabbit secondary antibody for 1 h at room temperature and stained with 3,3’-diaminobenzidine solution, and observed using Leica microscope.

### TUNEL assay

Cell apoptosis was detected using the TUNEL assay kit as previously described [68]. In brief, lung cancer cells were seeded on glass cover slips in a six-well plate at 50,000 cells per well and incubated for 48 h, and then fixed in a 4% paraformaldehyde solution for 20 min, permeabilized with 0.3% Triton X-100 for 10 min, washed for three times with PBS, and stained by the fresh TUNEL assay solution. After incubation in the dark at 37 °C for 60 min, the cells were incubated with DAPI for 10 min in the dark. The apoptotic cells were imaged using OLYMPUS FSX-100 confocal microscopy.

### Cell apoptosis assay

The cell apoptosis was assessed using 7-AAD and PE double staining method [68]. In brief, cells were incubated with cisplatin at concentrations of 0–30 μM, harvested, and then stained with 7-AAD and PE. Then, the stained cells were analyzed by flow cytometry.

### Luciferase assay

The genomic DNA fragments that have the length from −1600 bp to +400 bp in the 5′flanking promoter region of the *KRT7-AS* gene were cloned into the luciferase reporter vector pGL4.17. A549 cells were transfected with either pGL4.17-*KRT7-AS* promoter DNA or pGL4.17 vector control. The luciferase activity in cell lysates was measured using the Dual-luciferase Reporter assay kit as previously described [68].

### RNA Binding Protein Immunoprecipitation assay

RNA binding protein immunoprecipitation (RIP) assay was conducted as reported by Yang et al. [68]. Cultured lung cancer cells were collected and treated with 0.3% formaldehyde in PBS for 10 min, then incubated with 0.125 M glycine-PBS for 5 min at 22 °C. The cell pellets were re-suspended in RIP assay buffer for 30 min with shaking and then centrifuged at 4 °C, 1200 rpm for 15 min. The cleared lysates were incubated for 4 h at 4 °C with PTEN antibody. The pellets were washed, re-suspended, and treated with RIP assay buffer containing proteinase K at 45 °C for 45 min. Finally, RNA was isolated with TRIZOL RNA isolation kit, and KRT7-AS mRNA was detected with RT-PCR.

### In vitro transcription and RNA pulldown assay

Biotin-labeled full-length and truncated fragments of KRT7-AS RNA were transcribed in vitro with a Biotin RNA Labeling Mix Kit and T7 RNA polymerase using the KRT7-AS cDNA as a template [70]. The transcription products were isolated with a RNeasy Mini kit, and the folded RNA was mixed with cancer cell lysate and incubated at 22 °C for 2 h, then incubated with streptavidin agarose beads at 22 °C for 1 h. The beads were boiled in Laemmli loading buffer and samples were run on SDS-PAGE, and the RNA pulldown-proteins were analyzed by Western blotting.

### RNA fluorescence in situ hybridization assay

For RNA fluorescence in situ hybridization (FISH) assay, Cy3-labeled KRT7-AS probe was used to detect the non-coding RNA expression following the manufacturer’s instructions [71]. DAPI was used to indicate the nucleus. All images were obtained with OLYMPUS FSX-100 confocal microscopy.

### Lung and breast cancer patients and tumor samples

Ten paired breast cancer and adjacent normal tissues were obtained from the First Affiliated Hospital of Soochow University. Additionally, five paired lung cancer and adjacent non-tumorous tissues were obtained from the Dushu Lake Hospital Affiliated to Soochow University. The First Affiliated Hospital of Soochow University and the Dushu Lake Hospital Affiliated to Soochow University have approved our studies. All of the resected cancer samples were confirmed by clinical diagnostic pathology, and we have received informed consent from all subjects. No local or systemic neoadjuvant radiotherapy and/or chemotherapy, and targeted therapy were managed in the patients.

### RNA-Seq and gene expression profile analysis

RNA-Seq was performed as we previously reported [72]. In brief, the total RNAs in three samples of either KRT7-AS overexpressed H1299 lung cancer cells or three samples of vector control H1299 cells were extracted by Trizol solution (Invitrogen), respectively. The gene expression levels in these cells were detected using the Affymetrix Human Genome U133 Plus 2.0 Array (containing 48,000 transcripts, total 32,375 human genes, including cDNA controls) by the Shanghai Biotechnology Co., Ltd (Shanghai, China). The upregulated and downregulated genes with more or less than 3 folds (*p* < 0.05) in the KRT7-AS overexpressed H1299 cells as compared with the vector control H1299 cells were analyzed using Affy package in R language (v3.4.4). The enrichment analyses of gene ontology (GO) functional categories and KEGG pathways of differentially expressed genes (DEGs) were performed by DAVID tools as shown in the Supplementary Figs. S3 and S4.

### Statistical analysis

All results represent the mean ± SD. Differences between the groups were assessed by one-way ANOVA using GraphPad Prism 5. Statistical comparisons were performed using the Student’s *t*-test, and the significance of differences was indicated as *P < 0.05, **P < 0.01, and ***P < 0.001.

## Supplementary information

Supplemental figure 1

Supplemental figure 2

Supplemental figure 3

Supplemental figure 4

Supplemental figure 5

Supplemental figure 6

Supplemental figure legends

checklist

Original full length western blots

## Data Availability

Available by request from the corresponding author or from commercial sources when applicable.

## References

[1] Nagano T, Fraser P (2011). No-nonsense functions for long noncoding RNAs. Cell..

[2] Prensner JR, Chinnaiyan AM (2011). The emergence of lncRNAs in cancer biology. Cancer Discov..

[3] Statello L, Guo CJ, Chen LL, Huarte M (2021). Gene regulation by long non-coding RNAs and its biological functions. Nat Rev Mol Cell Biol..

[4] Evan GI, Vousden KH (2001). Proliferation, cell cycle and apoptosis in cancer. Nature..

[5] Wu J, Minikes AM, Gao M, Bian H, Li Y, Stockwell BR (2019). Intercellular interaction dictates cancer cell ferroptosis via NF2-YAP signalling. Nature..

[6] Medina CB, Mehrotra P, Arandjelovic S, Perry JSA, Guo Y, Morioka S (2020). Metabolites released from apoptotic cells act as tissue messengers. Nature..

[7] Chatterjee M, Viswanathan P (2021). Long noncoding RNAs in the regulation of p53-mediated apoptosis in human cancers. Cell Biol Int..

[8] Ghafouri-Fard S, Aghabalazade A, Shoorei H, Majidpoor J, Taheri M, Mokhtari M (2021). The impact of lncRNAs and miRNAs on apoptosis in lung cancer. Front Oncol..

[9] Erdogan I, Sweef O, Akgul B (2022). Long noncoding RNAs in human cancer and apoptosis. Curr Pharm Biotechnol.

[10] Ghaemi S, Fekrirad Z, Zamani N, Rahmani R, Arefian E (2022). Non-coding RNAs enhance the apoptosis efficacy of therapeutic agents used for the treatment of glioblastoma multiform. J Drug Target..

[11] Kadosh E, Snir-Alkalay I, Venkatachalam A, May S, Lasry A, Elyada E (2020). The gut microbiome switches mutant p53 from tumour-suppressive to oncogenic. Nature..

[12] Abadi AJ, Zarrabi A, Gholami MH, Mirzaei S, Hashemi F, Zabolian A (2021). Small in size, but large in action: microRNAs as potential modulators of PTEN in breast and lung cancers. Biomolecules..

[13] Nikkhoo A, Rostami N, Hojjat-Farsangi M, Azizi G, Yousefi B, Ghalamfarsa G (2019). Smac mimetics as novel promising modulators of apoptosis in the treatment of breast cancer. J Cell Biochem..

[14] Sordo-Bahamonde C, Lorenzo-Herrero S, Payer AR, Gonzalez S, Lopez-Soto A (2020). Mechanisms of apoptosis resistance to NK cell-mediated cytotoxicity in cancer. Int J Mol Sci..

[15] Balaji S, Terrero D, Tiwari AK, Ashby CR, Raman D (2021). Alternative approaches to overcome chemoresistance to apoptosis in cancer. Adv Protein Chem Struct Biol..

[16] Bozzato AM, Martingano P, Pozzi Mucelli RA, Cavallaro MFM, Cesarotto M, Marcello C (2022). MicroRNAs related to TACE treatment response: a review of the literature from a radiological point of view. Diagnostics..

[17] Huang B, Song JH, Cheng Y, Abraham JM, Ibrahim S, Sun Z (2016). Long non-coding antisense RNA KRT7-AS is activated in gastric cancers and supports cancer cell progression by increasing KRT7 expression. Oncogene..

[18] Chen S, Su T, Zhang Y, Lee A, He J, Ge Q (2020). Fusobacterium nucleatum promotes colorectal cancer metastasis by modulating KRT7-AS/KRT7. Gut Microbes..

[19] Chen F, Chen Z, Guan T, Zhou Y, Ge L, Zhang H (2021). N(6) -Methyladenosine regulates mRNA stability and translation efficiency of KRT7 to promote breast cancer lung metastasis. Cancer Res..

[20] Chan ES, Alexander J, Swanson PE, Jain D, Yeh MM (2012). PDX-1, CDX-2, TTF-1, and CK7: a reliable immunohistochemical panel for pancreatic neuroendocrine neoplasms. Am J Surg Pathol..

[21] Li Y, Su Z, Wei B, Liang Z (2021). KRT7 overexpression is associated with poor prognosis and immune cell infiltration in patients with pancreatic adenocarcinoma. Int J Gen Med..

[22] He Y, Yue H, Cheng Y, Ding Z, Xu Z, Lv C (2021). ALKBH5-mediated m(6)A demethylation of KCNK15-AS1 inhibits pancreatic cancer progression via regulating KCNK15 and PTEN/AKT signaling. Cell Death Dis..

[23] Yahyazadeh R, Bashash D, Ghaffari P, Kord S, Safaroghli-Azar A, Ghaffari SH (2021). Evaluation of hTERT, KRT7, and survivin in urine for noninvasive detection of bladder cancer using real-time PCR. BMC Urol.

[24] An Q, Liu T, Wang MY, Yang YJ, Zhang ZD, Liu ZJ (2021). KRT7 promotes epithelialmesenchymal transition in ovarian cancer via the TGFbeta/Smad2/3 signaling pathway. Oncol Rep..

[25] Zhang Z, Tu K, Liu F, Liang M, Yu K, Wang Y (2020). FoxM1 promotes the migration of ovarian cancer cell through KRT5 and KRT7. Gene..

[26] Wang P, Magdolen V, Seidl C, Dorn J, Drecoll E, Kotzsch M (2018). Kallikrein-related peptidases 4, 5, 6 and 7 regulate tumour-associated factors in serous ovarian cancer. Br J Cancer..

[27] Borziak K, Finkelstein J (2021). Comparative analysis of public data sets to identify stemness markers that differentiate liver cancer stem cells. Stud Health Technol Inform..

[28] Borziak K, Finkelstein J (2021). Identification of liver cancer stem cell stemness markers using a comparative analysis of public data sets. Stem Cells Cloning..

[29] Jiang M, Li H, Zhang Y, Yang Y, Lu R, Liu K (2017). Transitional basal cells at the squamous-columnar junction generate Barrett’s oesophagus. Nature..

[30] Koren A, Sodja E, Rijavec M, Jez M, Kovac V, Korosec P (2015). Prognostic value of cytokeratin-7 mRNA expression in peripheral whole blood of advanced lung adenocarcinoma patients. Cell Oncol..

[31] Nowicki-Osuch K, Zhuang L, Jammula S, Bleaney CW, Mahbubani KT, Devonshire G (2021). Molecular phenotyping reveals the identity of Barrett’s esophagus and its malignant transition. Science..

[32] Wu Y, Lv M, Qian T, Shen Y (2021). Correlation analysis of Ki67 and CK7 expression with clinical characteristics and prognosis of postoperative cervical adenocarcinoma patients. Ann Palliat Med.

[33] Roe OD, Szulkin A, Anderssen E, Flatberg A, Sandeck H, Amundsen T (2012). Molecular resistance fingerprint of pemetrexed and platinum in a long-term survivor of mesothelioma. PLoS ONE.

[34] Zhang X, Wang J, Lu J, Su L, Wang C, Huang Y (2021). Robust prognostic subtyping of muscle-invasive bladder cancer revealed by deep learning-based multi-omics data integration. Front Oncol.

[35] Dixon K, Brew T, Farnell D, Godwin TD, Cheung S, Chow C (2021). Modelling hereditary diffuse gastric cancer initiation using transgenic mouse-derived gastric organoids and single-cell sequencing. J Pathol.

[36] Loupakis F, Biason P, Prete AA, Cremolini C, Pietrantonio F, Pella N (2019). CK7 and consensus molecular subtypes as major prognosticators in (V600E)BRAF mutated metastatic colorectal cancer. Br J Cancer.

[37] Czapiewski P, Bobowicz M, Peksa R, Skrzypski M, Gorczynski A, Szczepanska-Michalska K (2016). Keratin 7 expression in lymph node metastases but not in the primary tumour correlates with distant metastases and poor prognosis in colon carcinoma. Pol J Pathol.

[38] Gkountakos A, Sartori G, Falcone I, Piro G, Ciuffreda L, Carbone C (2019). PTEN in lung cancer: dealing with the problem, building on new knowledge and turning the game around. Cancers.

[39] Alvarez-Garcia V, Tawil Y, Wise HM, Leslie NR (2019). Mechanisms of PTEN loss in cancer: It’s all about diversity. Semin Cancer Biol.

[40] Wang Q, Wang J, Xiang H, Ding P, Wu T, Ji G (2021). The biochemical and clinical implications of phosphatase and tensin homolog deleted on chromosome ten in different cancers. Am J Cancer Res.

[41] Chai C, Wu HH, Abuetabh Y, Sergi C, Leng R (2022). Regulation of the tumor suppressor PTEN in triple-negative breast cancer. Cancer Lett.

[42] Li ZH, Li L, Kang LP, Wang Y (2018). MicroRNA-92a promotes tumor growth and suppresses immune function through activation of MAPK/ERK signaling pathway by inhibiting PTEN in mice bearing U14 cervical cancer. Cancer Med.

[43] Shin JW, Kim SH, Yoon JY (2021). PTEN downregulation induces apoptosis and cell cycle arrest in uterine cervical cancer cells. Exp Ther Med.

[44] Kwon S, Chan AT (2020). Extracting the benefits of berberine for colorectal cancer. Lancet Gastroenterol Hepatol.

[45] Yuan C, Wu M, Xiao Q, Zhao W, Li H, Zhong Y (2021). Blocking Msr1 by berberine alkaloids inhibits caspase-11-dependent coagulation in bacterial sepsis. Signal Transduct Target Ther.

[46] Zaurin R, Ferrari R, Nacht AS, Carbonell J, Le Dily F, Font-Mateu J (2021). A set of accessible enhancers enables the initial response of breast cancer cells to physiological progestin concentrations. Nucleic Acids Res.

[47] Wen W, Chen Z, Bao J, Long Q, Shu XO, Zheng W (2021). Genetic variations of DNA bindings of FOXA1 and co-factors in breast cancer susceptibility. Nat Commun.

[48] Yanagi S, Kishimoto H, Kawahara K, Sasaki T, Sasaki M, Nishio M (2007). Pten controls lung morphogenesis, bronchioalveolar stem cells, and onset of lung adenocarcinomas in mice. J Clin Investig.

[49] Cai J, Li R, Xu X, Zhang L, Lian R, Fang L (2018). CK1alpha suppresses lung tumour growth by stabilizing PTEN and inducing autophagy. Nat Cell Biol.

[50] Wang X, Trotman LC, Koppie T, Alimonti A, Chen Z, Gao Z (2007). NEDD4-1 is a proto-oncogenic ubiquitin ligase for PTEN. Cell..

[51] Chalhoub N, Baker SJ (2009). PTEN and the PI3-kinase pathway in cancer. Annu Rev Pathol.

[52] Amodio N, Scrima M, Palaia L, Salman AN, Quintiero A, Franco R (2010). Oncogenic role of the E3 ubiquitin ligase NEDD4-1, a PTEN negative regulator, in non-small-cell lung carcinomas. Am J Pathol.

[53] Maddika S, Kavela S, Rani N, Palicharla VR, Pokorny JL, Sarkaria JN (2011). WWP2 is an E3 ubiquitin ligase for PTEN. Nat Cell Biol.

[54] Li H, Zhang P, Zhang Q, Li C, Zou W, Chang Z (2018). WWP2 is a physiological ubiquitin ligase for phosphatase and tensin homolog (PTEN) in mice. J Biol Chem.

[55] Lyu J, Yu X, He L, Cheng T, Zhou J, Cheng C (2015). The protein phosphatase activity of PTEN is essential for regulating neural stem cell differentiation. Mol Brain.

[56] Dempsey DR, Viennet T, Iwase R, Park E, Henriquez S, Chen Z (2021). The structural basis of PTEN regulation by multi-site phosphorylation. Nat Struct Mol Biol.

[57] Kryczka J, Czarnecka-Chrebelska KH, Brzezianska-Lasota E (2021). Molecular mechanisms of chemoresistance induced by cisplatin in NSCLC cancer therapy. Int J Mol Sci.

[58] Li C, Yang N, Chen Z, Xia N, Shan Q, Wang Z (2021). Hypoxia-induced Tie1 drives stemness and cisplatin resistance in non-small cell lung carcinoma cells. Cancer Cell Int.

[59] Tella SH, Kommalapati A, Borad MJ, Mahipal A (2020). Second-line therapies in advanced biliary tract cancers. Lancet Oncol.

[60] Zhang Y, Chen L, Hu GQ, Zhang N, Zhu XD, Yang KY (2019). Gemcitabine and cisplatin induction chemotherapy in nasopharyngeal carcinoma. N Engl J Med.

[61] Dasari S, Tchounwou PB (2014). Cisplatin in cancer therapy: molecular mechanisms of action. Eur J Pharmacol.

[62] Galluzzi L, Senovilla L, Vitale I, Michels J, Martins I, Kepp O (2012). Molecular mechanisms of cisplatin resistance. Oncogene..

[63] Chovanec M, Abu Zaid M, Hanna N, El-Kouri N, Einhorn LH, Albany C (2017). Long-term toxicity of cisplatin in germ-cell tumor survivors. Ann Oncol.

[64] Fennell DA, Danson S, Woll PJ, Forster M, Talbot D, Child J (2020). Ganetespib in combination with pemetrexed-platinum chemotherapy in patients with pleural mesothelioma (MESO-02): a phase Ib trial. Clin Cancer Res.

[65] Papadimitrakopoulou VA, Mok TS, Han JY, Ahn MJ, Delmonte A, Ramalingam SS (2020). Osimertinib versus platinum-pemetrexed for patients with EGFR T790M advanced NSCLC and progression on a prior EGFR-tyrosine kinase inhibitor: AURA3 overall survival analysis. Ann Oncol.

[66] Yu X, Wang S, Wang J, Gong J, Shi J, Yu S (2020). Berberine induces CYP2J2 expression in human U251 glioma cells via regulation of peroxisome proliferator-activated receptor alpha. Pharmacology..

[67] Zhao Z, Xiang S, Qi J, Wei Y, Zhang M, Yao J (2020). Correction of the tumor suppressor Salvador homolog-1 deficiency in tumors by lycorine as a new strategy in lung cancer therapy. Cell Death Dis.

[68] Yang B, Zhang B, Cao Z, Xu X, Huo Z, Zhang P (2020). The lipogenic LXR-SREBF1 signaling pathway controls cancer cell DNA repair and apoptosis and is a vulnerable point of malignant tumors for cancer therapy. Cell Death Differ.

[69] Xiang S, Zhao Z, Zhang T, Zhang B, Meng M, Cao Z (2020). Triptonide effectively suppresses gastric tumor growth and metastasis through inhibition of the oncogenic Notch1 and NF-kappaB signaling pathways. Toxicol Appl Pharmacol.

[70] Li RH, Tian T, Ge QW, He XY, Shi CY, Li JH (2021). A phosphatidic acid-binding lncRNA SNHG9 facilitates LATS1 liquid-liquid phase separation to promote oncogenic YAP signaling. Cell Res.

[71] Zhang Y, Li Y, Hu Q, Xi Y, Xing Z, Zhang Z (2020). The lncRNA H19 alleviates muscular dystrophy by stabilizing dystrophin. Nat Cell Biol.

[72] Shang B, Gao A, Pan Y, Zhang G, Tu J, Zhou Y (2014). CT45A1 acts as a new proto-oncogene to trigger tumorigenesis and cancer metastasis. Cell Death Dis.

