# Supplementary figures and images for: The long non-coding RNA keratin-7 antisense acts as a new tumor suppressor to inhibit tumorigenesis and enhance apoptosis in lung and breast cancers
Source: Cell Death Dis. 2023 Apr 25;14(4):293. doi: 10.1038/s41419-023-05802-3 (PMC10130017; doi:10.1038/s41419-023-05802-3)

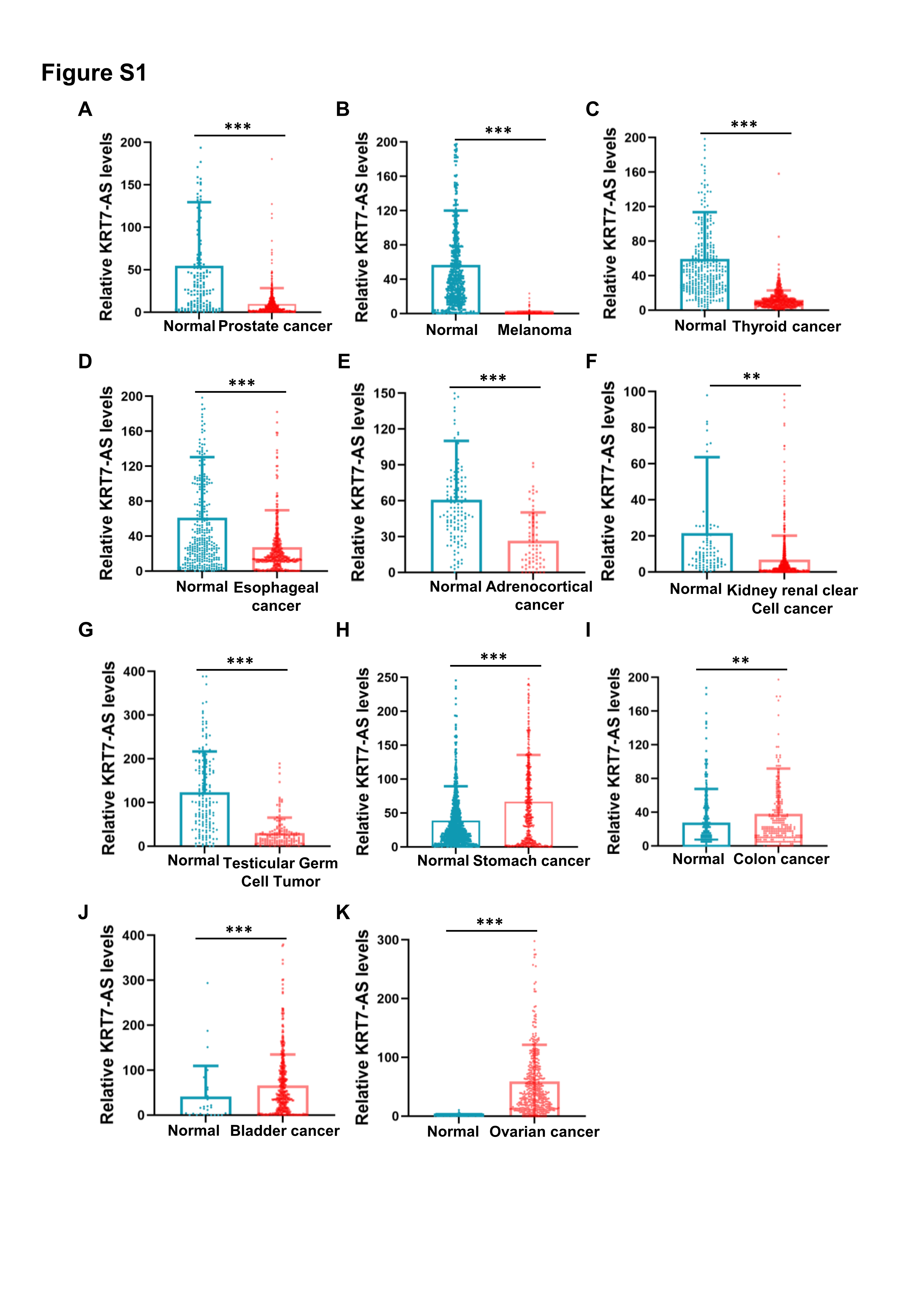

Supplement: Supplementary file 1 — Supplemental figure 1 [file 41419_2023_5802_MOESM1_ESM.tif]

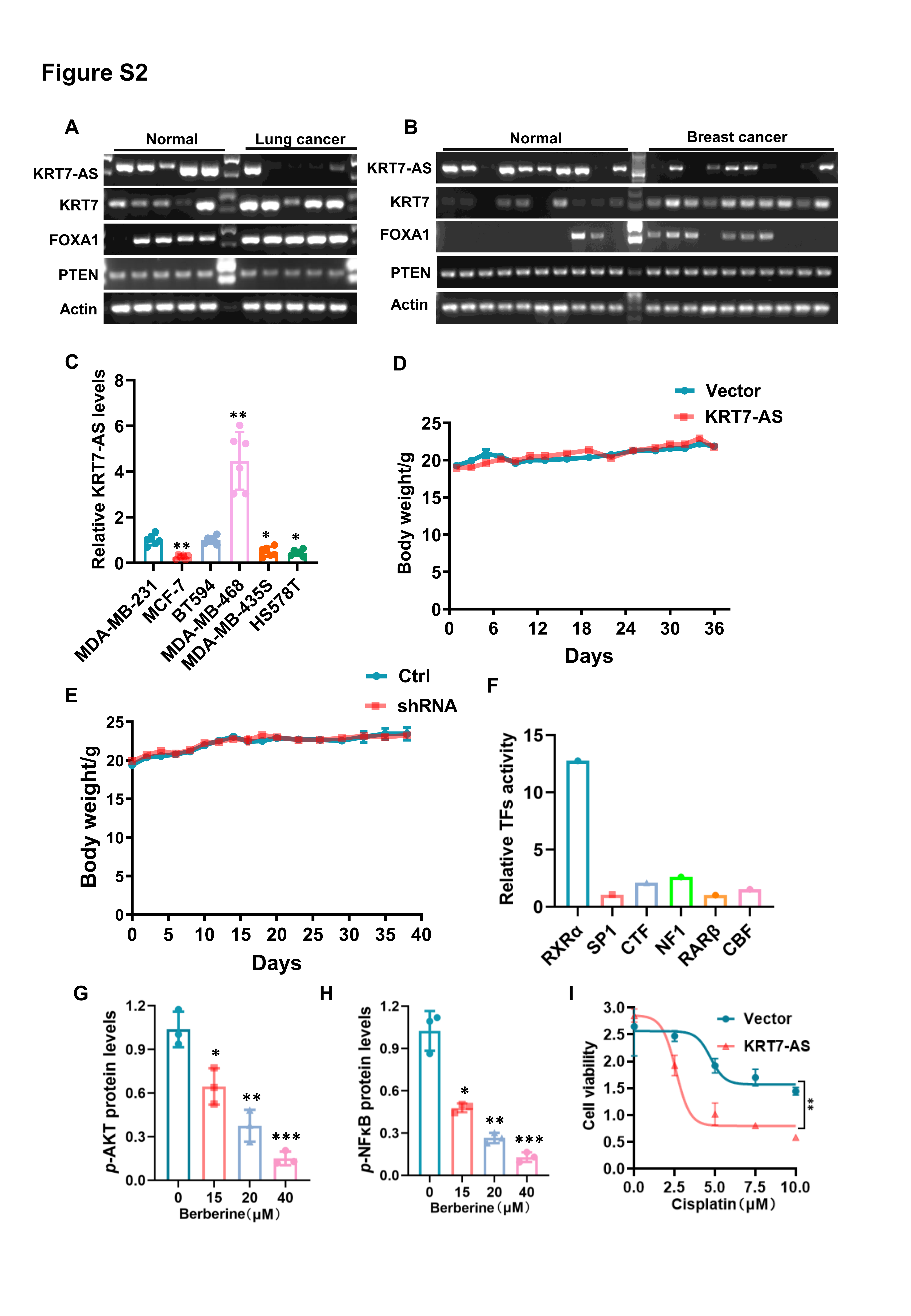

Supplement: Supplementary file 2 — Supplemental figure 2 [file 41419_2023_5802_MOESM2_ESM.tif]

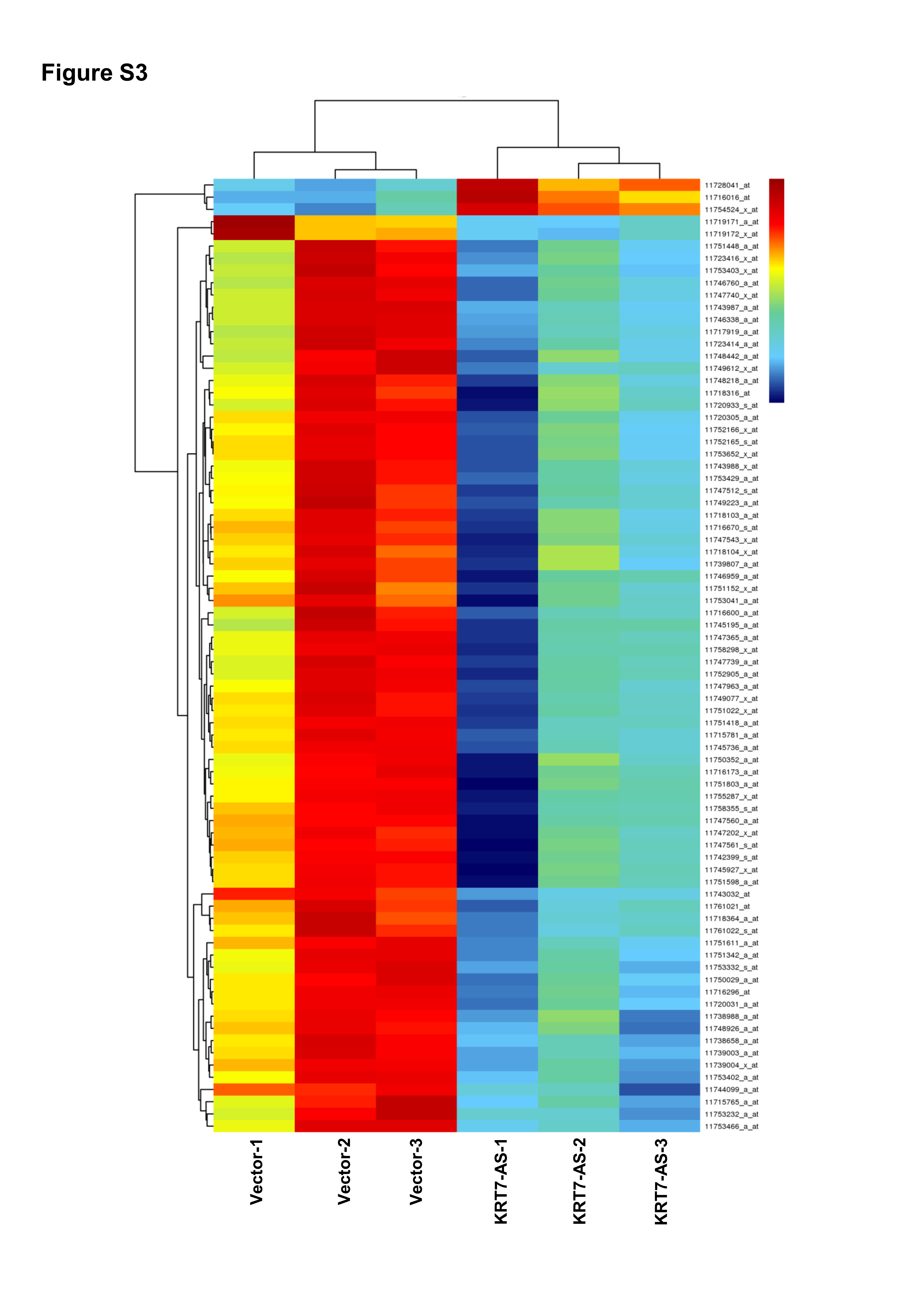

Supplement: Supplementary file 3 — Supplemental figure 3 [file 41419_2023_5802_MOESM3_ESM.tif]

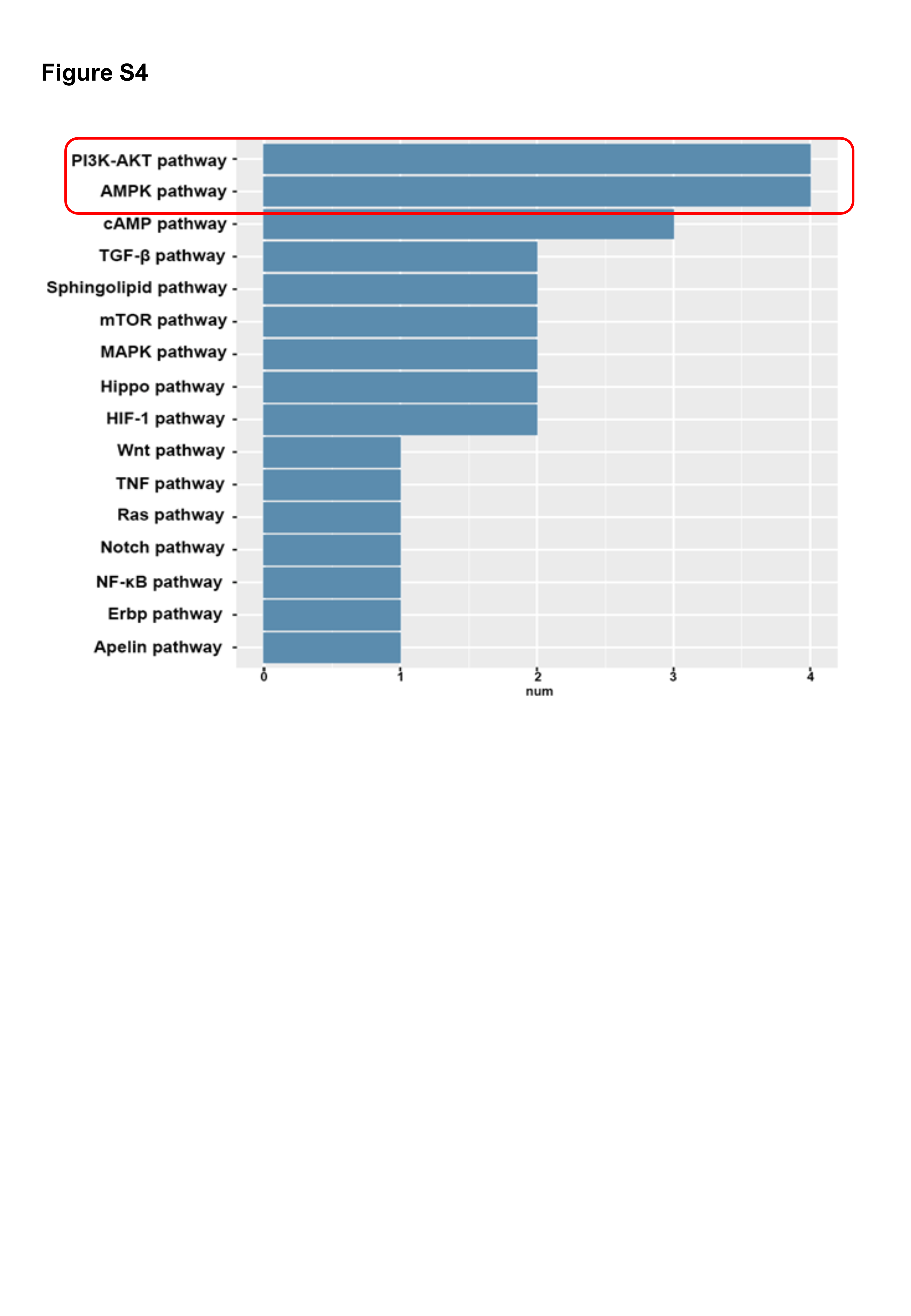

Supplement: Supplementary file 4 — Supplemental figure 4 [file 41419_2023_5802_MOESM4_ESM.tif]

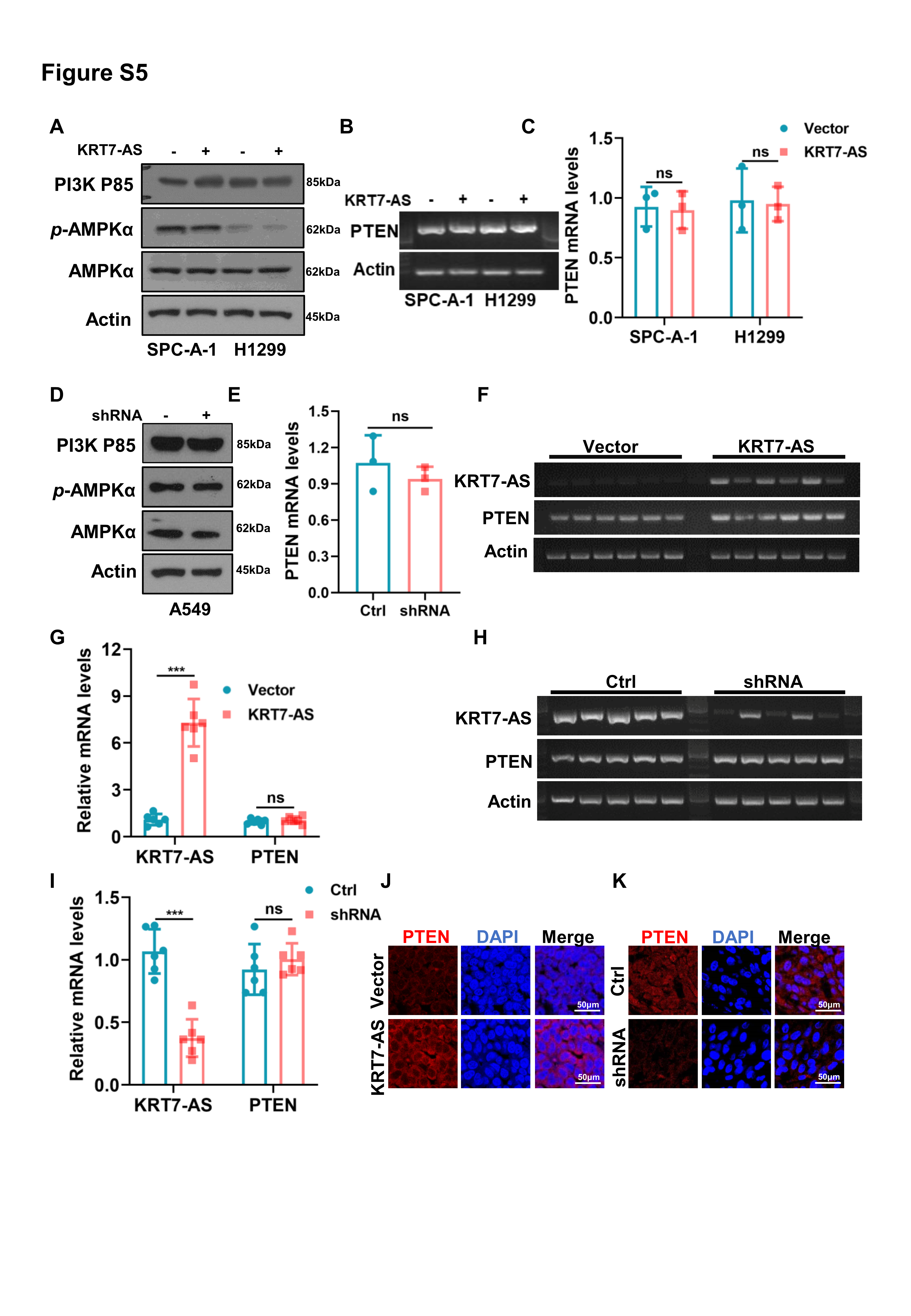

Supplement: Supplementary file 5 — Supplemental figure 5 [file 41419_2023_5802_MOESM5_ESM.tif]

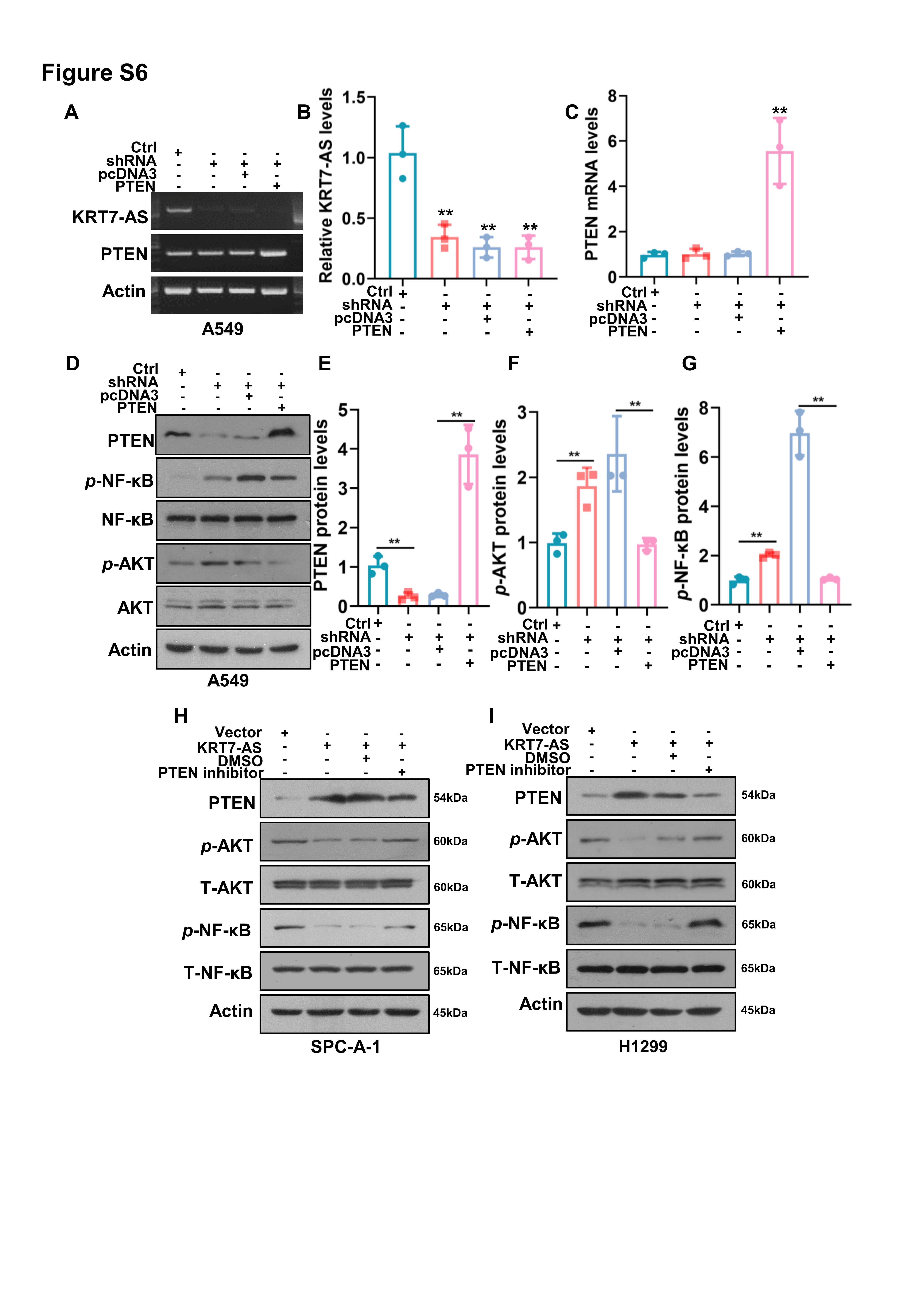

Supplement: Supplementary file 6 — Supplemental figure 6 [file 41419_2023_5802_MOESM6_ESM.tif]
