# Supplementary material for: The long non-coding RNA keratin-7 antisense acts as a new tumor suppressor to inhibit tumorigenesis and enhance apoptosis in lung and breast cancers
Source: Cell Death Dis. 2023 Apr 25;14(4):293. doi: 10.1038/s41419-023-05802-3 (PMC10130017; doi:10.1038/s41419-023-05802-3)
Supplement: Supplementary file 7 — Supplemental figure legends [file 41419_2023_5802_MOESM7_ESM.docx]

**Supplementary Figure legends**

**Supplementary Figure S1. Expression of the lncRNA KRT7-AS in 33 types of cancers.**

We mined the TCGA database to check *KRT7-AS* gene expression in 33 types of human cancer and found that KRT7-AS was aberrantly overexpressed in prostate cancer (A), melanoma (B), thyroid cancer (C), esophageal cancer (D), adrenocortical cancer (E), kidney renal clear cell cancer (F), testicular germ cell tumor (G), stomach cancer (H), colon cancer (I), bladder cancer (J), ovarian cancer (K), and the adjacent normal tissues were as a control. Of note, KRT7-AS was also abnormally overexpressed in lung cancer and breast cancer as depictured in the main Figure 1A and 1B. The data are processed by integrative analysis. ** P<0.01, *** P<0.001.

**Supplementary Figure S2.** **The expression and function of KRT7-AS, FOXA1, and PTEN in lung cancer and breast cancer tissues and cells.**

The mRNA levels of KRT7-AS, KRT7, FOXA1, and PTEN in paired clinical lung cancer tissues (A) and breast cancer tissues (B) were analyzed by RT-PCR. KRT7-AS levels were detected in six breast cancer cell lines, including MDA-MB-231, MCF-7, BT594, MDA-MB-468, MDA-MB-435S, and HS578T (C). The body weight of xenograft mice was quantified using GraphPad (D, E). LASAGNA method indicated that the *KRT7-AS* DNA fragment from -1600 to -1350 has six putative motifs for the binding of six transcription factors, and the RXRα possessed the highest activity score among these transcription factors (F). The RXRα agonist berberine decreased the phosphorylation of AKT and NF-κB (G, H). CTG assay showed KRT7-AS enhanced cisplatin sensibility in MCF-7 cells (I). Data are shown as mean ± SD of three independent replicates. *** P<0.001.

**Supplementary Figure S3. Analysis of gene expression profile in lung cancer cells using RNA-seq**

The gene expression profile of three samples from KRT7-AS overexpress lung cancer cells and three samples from control cells were depictured in S3. RNA-seq showed that overexpression of KRT7-AS in three samples of H1299 lung cancer cells (KRT7-AS-1, KRT7-AS-2, and KRT7-AS-3) resulted in marked change of the transcription levels of 85 genes (more or less than 3 folds, *p*<0.05) compared to those in vector control H1299 lung cancer cells (vector-1, vector-2, and vector-3) which barely express KRT7-AS. The red color means upregulation, the blue color means downregulation.

**Supplementary Figure S4. KEGG analysis of signaling pathways in KRT7-AS-overexpressed lung cancer cells.**

The enrichment analyses of KEGG pathways according to differentially expressed genes (DEGs) were performed by DAVID tools. The top 16 signal pathways were listed as shown in S4. The front two signaling pathways affected by KRT7-AS were indicated by a red frame.

**Supplementary Figure S5. KRT7-AS elevates the levels of tumor-suppressive PTEN in lung cancer cells.**

The effect of either overexpression of KRT7-AS or silencing of KRT7-AS on PI3KP85, *p*-AMPK, and AMPK levels in SPC-A-1 cells (A) and A549 lung cancer cells (D) was accessed by Western blotting. The PTEN mRNA levels in SPC-A-1, H1299, and A549 lung cancer cells (B, C, E) were detected by RT-PCR and real time qPCR, respectively. RT-PCR and qPCR showed that KRT7-AS did not affect PTEN mRNA levels in xenograft mice (F-I). IF indicated that PTEN was upregulated in KRT7-AS-overexpressed lung tissues from xenograft mice (J); whereas, silencing of KRT7-AS using shRNA obviously reduced PTEN levels (K). Data are shown as mean ± SD of three independent replicates. *** P<0.001.

**Supplementary Figure S6. KRT7-AS suppresses AKT and NF-κB signal pathways via PTEN.**

RT-PCR and real time qPCR showed that PTEN was re-overexpressed by transfection of KRT7-AS-silenced A549 cells with PTEN cDNA-pcDNA3 plasmid (A-C). Western blotting indicated that KRT7-AS shRNA-mediated elevation of *p*-AKT and *p*-NF-κB levels in lung cancer cells could be reversed by overexpression of PTEN (D-G). The levels of phosphorylated AKT and NF-κB in KRT7-AS-overexpressed lung cancer cells were tested by Western blotting after the cells were treated with the PTEN inhibitor VO-Ohpic trihydrate (H, I). Data are shown as mean ± SD of three independent replicates. ** P<0.01.
