# Supplementary material for: The long non-coding RNA keratin-7 antisense acts as a new tumor suppressor to inhibit tumorigenesis and enhance apoptosis in lung and breast cancers
Source: Cell Death Dis. 2023 Apr 25;14(4):293. doi: 10.1038/s41419-023-05802-3 (PMC10130017; doi:10.1038/s41419-023-05802-3)
Supplement: Supplementary file 9 — Original full length western blots [file 41419_2023_5802_MOESM9_ESM.docx]

**Uncropped original western blots**

**For Fig.3**

**
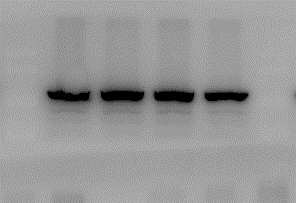

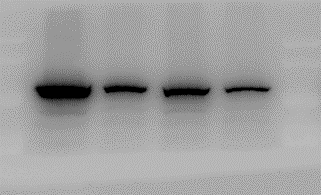
3G**

RxRα Actin


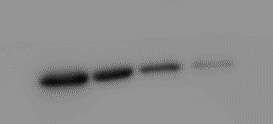
**
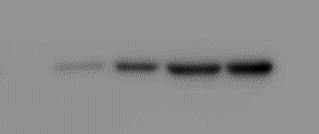
3J**

PTEN *p*-NF-kB

**
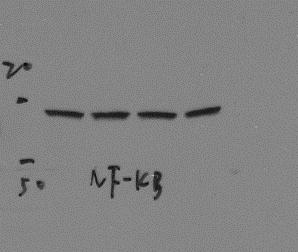

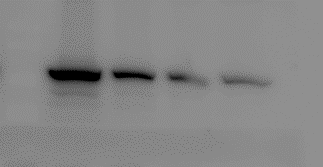
**

*p*-AKT T-NF-kB

**


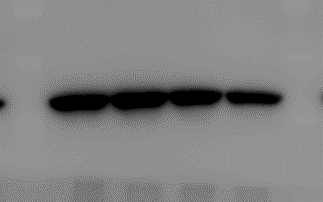
**

T-AKT Actin

**For Fig.4**

**4K**

**
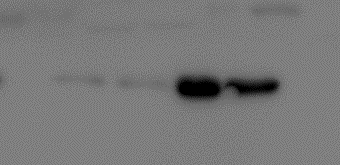

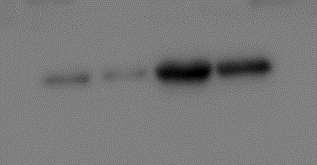
**

γ-H2AX

**
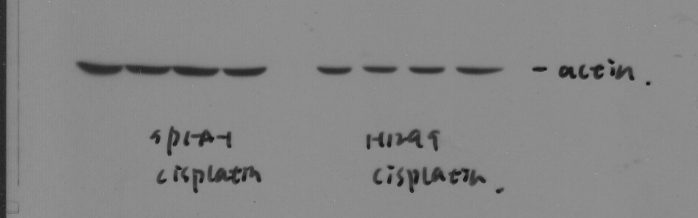

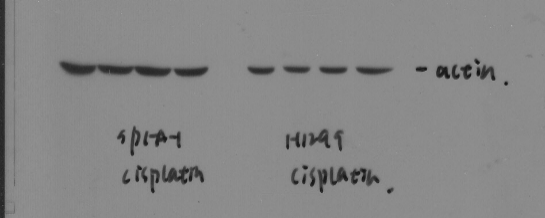
**

Actin

**
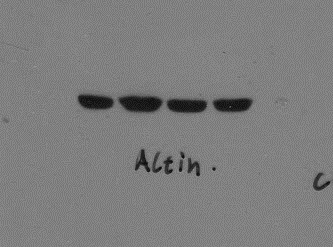


4L**

γ-H2AX Actin

**4M**

**
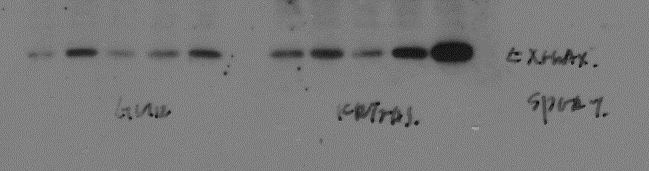


**γ-H2AX Actin

**
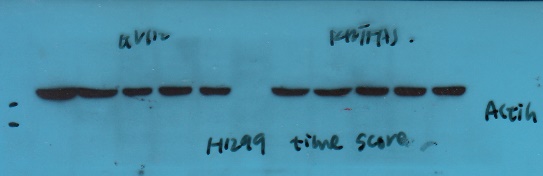

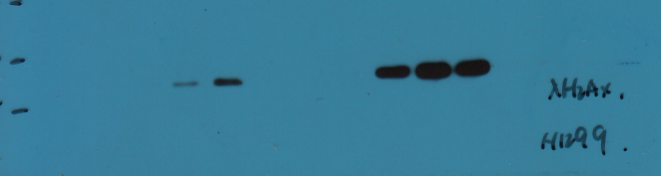
4N** γ-H2AX Actin

**



4O** γ-H2AX Actin

**For Fig.5**

**


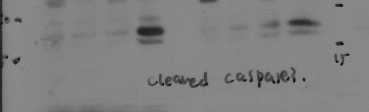

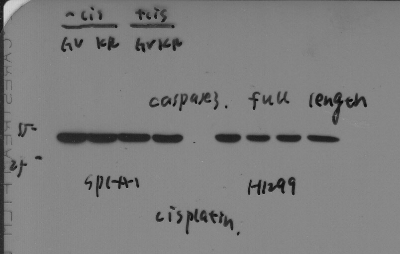
5D**  Caspase3 Cle-caspase Bcl-2

**
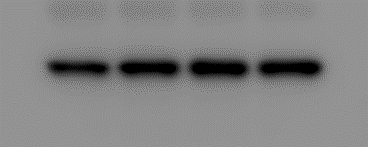

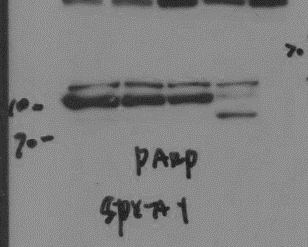
** PARP Actin

**
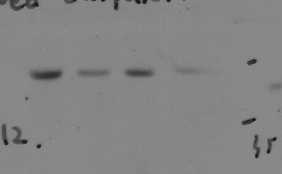

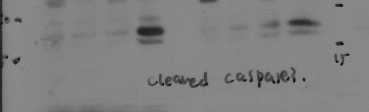

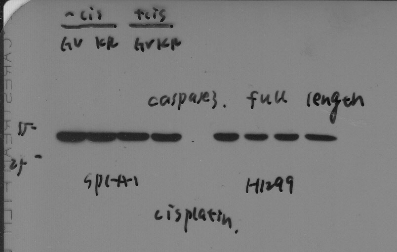
5E**  Caspase3 Cle-caspase Bcl-2

**
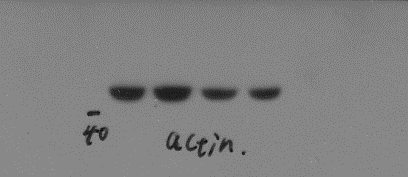

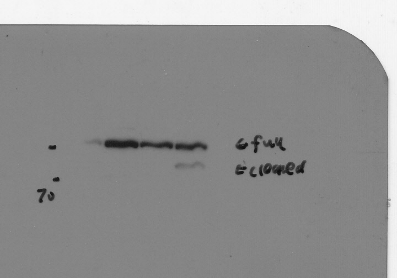
** PARP Actin

**




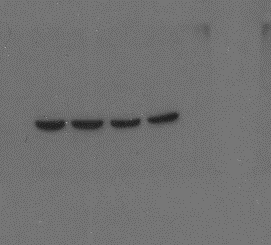
5F** Caspase3 Cle-caspase Bcl-2 PARP

**

**

**

**  Actin

**5G**


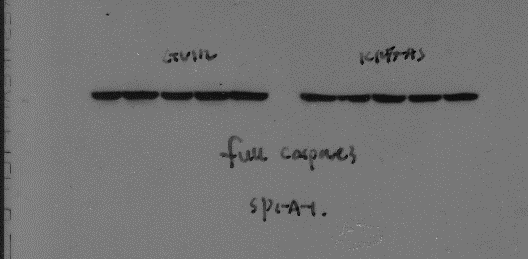

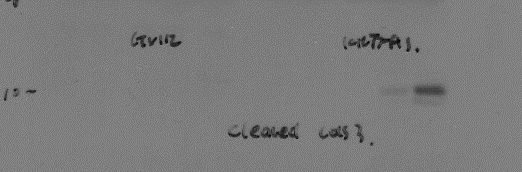
Caspase3 Cle-caspase3


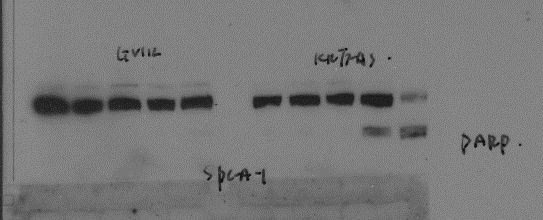
**
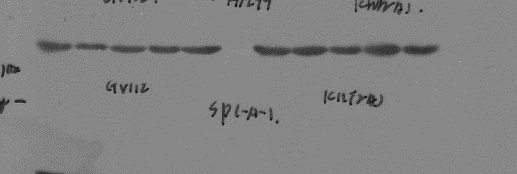
** PARP Actin

**5I**

**
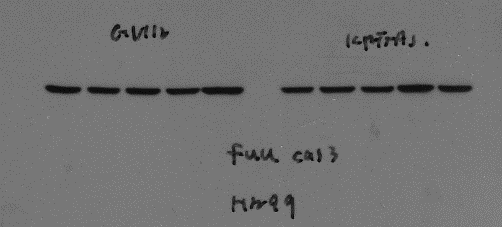

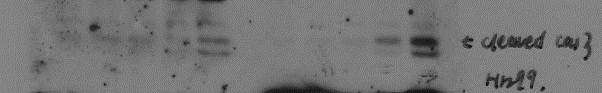
**Caspase3 Cle-caspase3

**
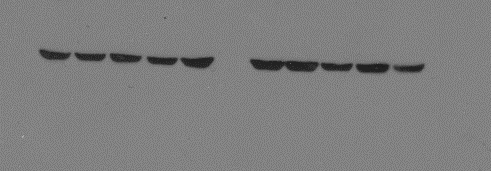

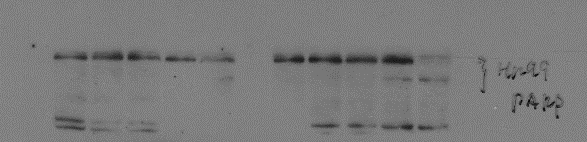
** PARP Actin

**5K**

**
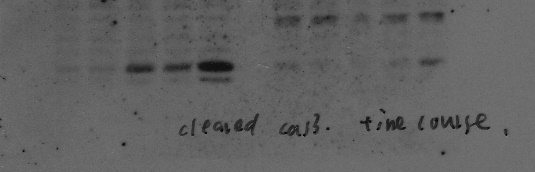

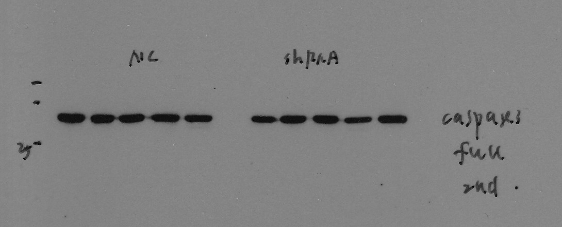
**Caspase3 Cle-caspase3

**



**PARP Bcl-2

**
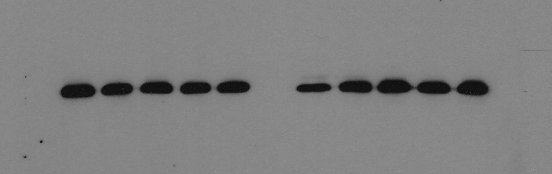
** Actin

**For Fig.6**

**
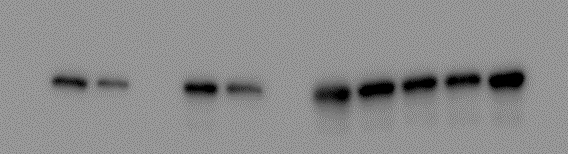

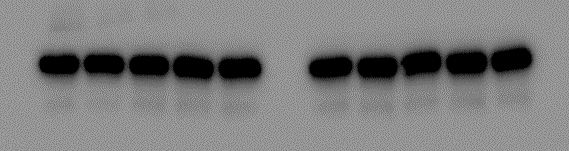
6B**  KRT7 Actin

**
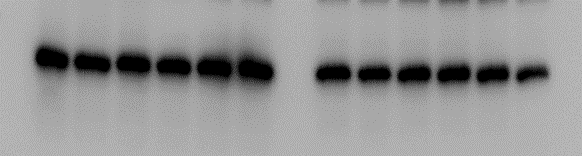

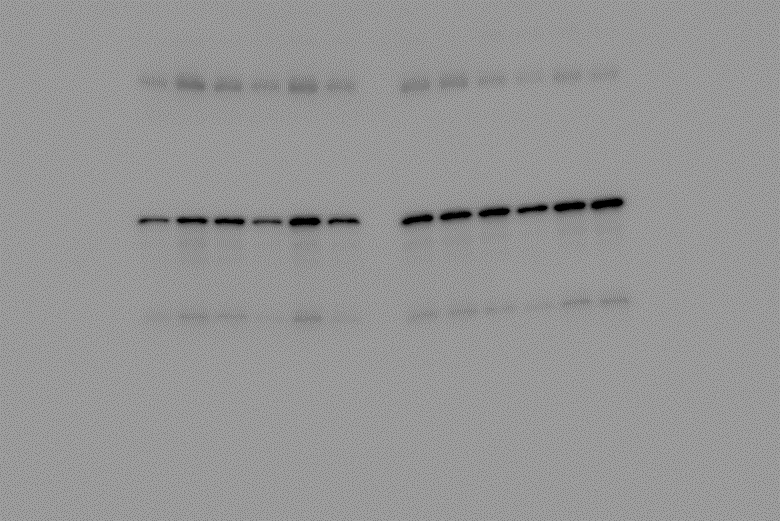
6C**  KRT7 Actin

**



6J**  KRT7 Actin

**For Fig.7**

**
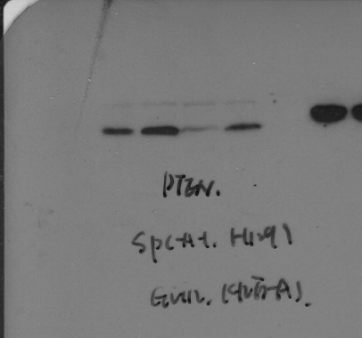




**
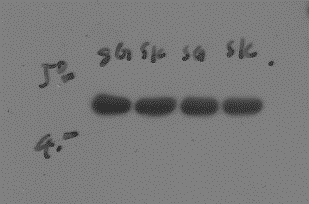
**7A** PTEN Actin **7B** PTEN Actin

**
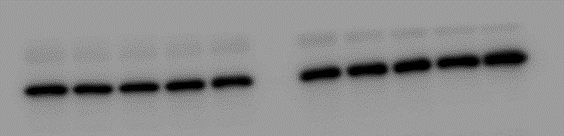

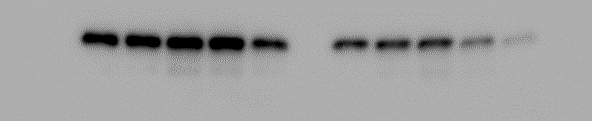
7E**  PTEN Actin

**7F**  PTEN Actin

**
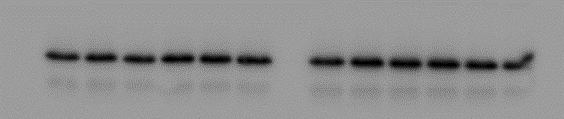


**

**



7K**  PTEN Actin

**
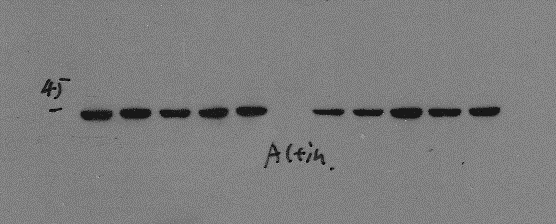

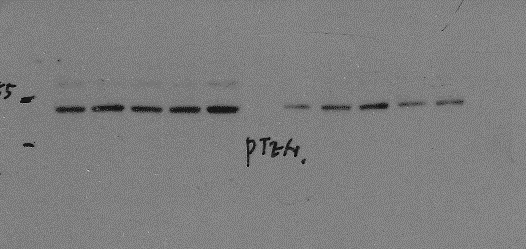
7L**  PTEN Actin

**For Fig.8**

**
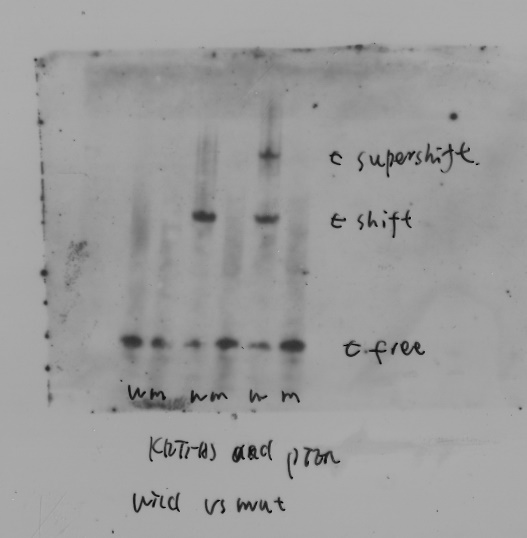
**

**For Fig.9**

**9A** PTEN Actin

**
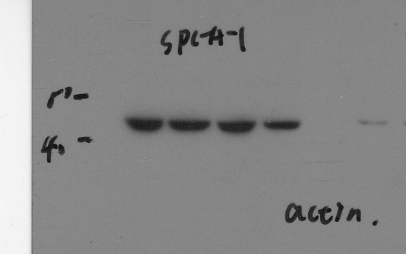

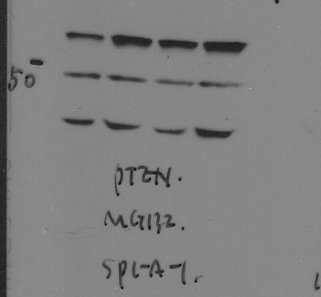
**

**9B** PTEN Actin

**
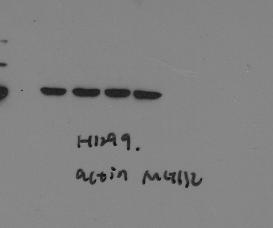

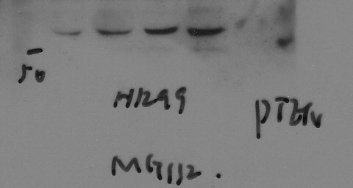
**

**9C** PTEN PTEN

**



**

**
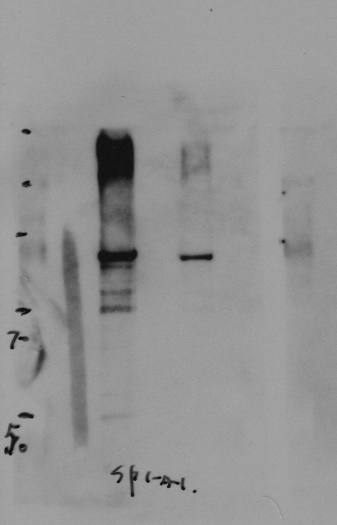

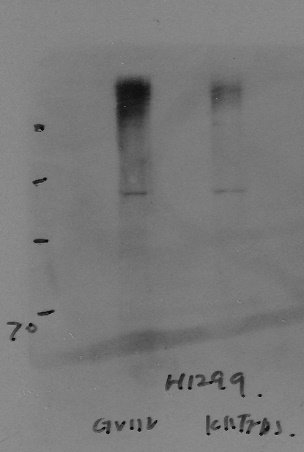
**UB UB

**9E** PTEN Actin

**
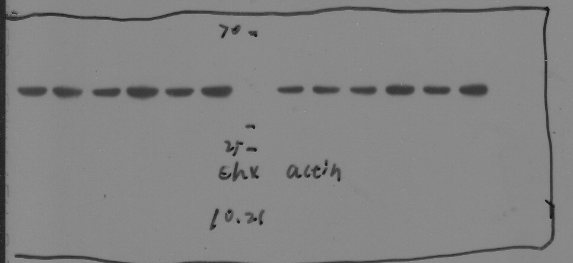

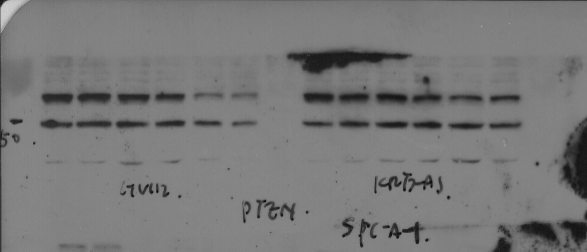
**

**9F** PTEN Actin

**
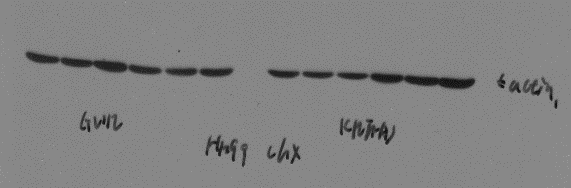

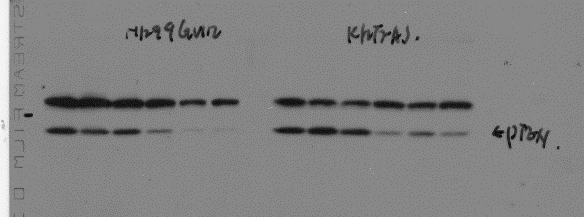
**

**9G** PTEN Actin

**



**

**9K** PTEN Actin

**


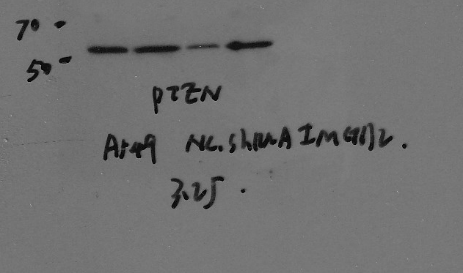
**

**



9M** PTEN UB
